# Genetic architecture of *maize chlorotic mottle virus* and maize lethal necrosis through GWAS, linkage analysis and genomic prediction in tropical maize germplasm

**DOI:** 10.1007/s00122-019-03360-x

**Published:** 2019-05-16

**Authors:** Chelang’at Sitonik, L. M. Suresh, Yoseph Beyene, Michael S. Olsen, Dan Makumbi, Kiplagat Oliver, Biswanath Das, Jumbo M. Bright, Stephen Mugo, Jose Crossa, Amsal Tarekegne, Boddupalli M. Prasanna, Manje Gowda

**Affiliations:** 1 International Maize and Wheat Improvement Center (CIMMYT), P.O. Box 1041-00621, Village Market, Nairobi, 00621 Kenya; 2 International Maize and Wheat Improvement Center (CIMMYT), El Batan, Texcoco, DF Mexico; 3 Department of Plant Breeding and Biotechnology, University of Eldoret (UoE), P.O. Box 1125, Eldoret, 30100 Kenya; 4 International Maize and Wheat Improvement Center (CIMMYT), 12.5 km Peg Mazowe Road, Mount Pleasant, P.O. Box MP163, Harare, Zimbabwe

## Abstract

**Key message:**

Analysis of the genetic architecture of MCMV and MLN resistance in maize doubled-haploid populations revealed QTLs with major effects on chromosomes 3 and 6 that were consistent across genetic backgrounds and environments. Two major-effect QTLs, *qMCMV3*-*108/qMLN3*-*108* and *qMCMV6*-*17/qMLN6*-*17*, were identified as conferring resistance to both MCMV and MLN.

**Abstract:**

Maize lethal necrosis (MLN) is a serious threat to the food security of maize-growing smallholders in sub-Saharan Africa. The ability of the *maize chlorotic mottle virus* (MCMV) to interact with other members of the Potyviridae causes severe yield losses in the form of MLN. The objective of the present study was to gain insights and validate the genetic architecture of resistance to MCMV and MLN in maize. We applied linkage mapping to three doubled-haploid populations and a genome-wide association study (GWAS) on 380 diverse maize lines. For all the populations, phenotypic variation for MCMV and MLN was significant, and heritability was moderate to high. Linkage mapping revealed 13 quantitative trait loci (QTLs) for MCMV resistance and 12 QTLs conferring MLN resistance. One major-effect QTL, *qMCMV3*-*108/qMLN3*-*108*, was consistent across populations for both MCMV and MLN resistance. Joint linkage association mapping (JLAM) revealed 18 and 21 main-effect QTLs for MCMV and MLN resistance, respectively. Another major-effect QTL, *qMCMV6*-*17/qMLN6*-*17*, was detected for both MCMV and MLN resistance. The GWAS revealed a total of 54 SNPs (MCMV-13 and MLN-41) significantly associated (*P* ≤ 5.60 × 10^−05^) with MCMV and MLN resistance. Most of the GWAS-identified SNPs were within or adjacent to the QTLs detected through linkage mapping. The prediction accuracy for within populations as well as the combined populations is promising; however, the accuracy was low across populations. Overall, MCMV resistance is controlled by a few major and many minor-effect loci and seems more complex than the genetic architecture for MLN resistance.

**Electronic supplementary material:**

The online version of this article (10.1007/s00122-019-03360-x) contains supplementary material, which is available to authorized users.

## Introduction

*Maize chlorotic mottle virus* (MCMV) is one of the most destructive pathogens, and it interacts synergistically with many members of the Potyviridae family: the potyviruses *sugarcane mosaic virus* (SCMV), *maize dwarf mosaic virus* (MDMV), and *wheat streak mosaic virus* (WSMV), resulting in maize lethal necrosis (MLN) (Wangai et al. 2012; Braidwood et al. 2018; Redinbaugh and Lucy 2018). MCMV is member of the genus *Machlomovirus* in the family Tombusviridae (Stenger and French 2008) and closely related to members of the genus *Carmovirus* (Wang et al. 2017; Redinbaugh and Lucy 2018). MCMV was first identified in Peru in 1974 and thereafter was reported in the USA, Brazil, Argentina, Mexico, Thailand, Hawaii and Colombia (Nelson et al. 2011).

Since 2010, MCMV has emerged at several locations around the world including China (Xie et al. 2011), Taiwan (Deng et al. 2014), Ecuador (Quito-Avila et al. 2016), and Spain (Braidwood et al. 2018). In sub-Saharan Africa (SSA), MCMV as one of the causal agents of MLN was first reported in Kenya (Wangai et al. 2012). Subsequently, both MCMV and MLN were reported in Tanzania, the Democratic Republic of Congo (Lukanda et al. 2014), Rwanda (Adams et al. 2014), Ethiopia and Uganda (Mahuku et al. 2015), resulting in significant yield loss and affecting the food security and livelihoods of smallholder farmers in eastern and central Africa. In eastern Africa, MCMV was found in co-infections with SCMV that cause MLN (Gowda et al. 2015; Beyene et al. 2017).

Understanding the genetic architecture of MCMV and MLN resistance is crucial in developing improved maize varieties with MLN resistance in SSA. Genome-wide association study (GWAS) and linkage-based mapping are two of the widely used approaches for identification of genomic regions influencing target traits in maize. Linkage-based mapping utilizes recombination events and marker–trait associations in biparental populations. This approach is powerful in capturing major genes with large-effect loci and rare alleles (Holland 2007; Semagn et al. 2010). However, resolving small-effect QTLs is challenging and the mapping resolution is comparatively low and typically produces large confidence intervals (Zhu et al. 2008; Li et al. 2010). In contrast, GWAS explores historical recombinations and functional variations within a huge set of individuals (Zhu et al. 2008; Yan et al. 2011). This is achieved through linkage disequilibrium (LD) analysis. Association mapping offers better resolution and greater ability to identify the favorable genetic loci responsible for the trait of interest (Flint-Garcia et al. 2005; Yu and Buckler 2006; Soto-Cerda and Cloutier 2012). GWAS is cost-effective and time-efficient because there is no need to generate a specific mapping population. GWAS has been successfully applied to identify genomic regions conferring resistance to important diseases of maize, such as *Fusarium* ear rot (Zila et al. 2013; Chen et al. 2016), maize rough dwarf disease (Chen et al. 2015), gray leaf spot (Shi et al. 2014), head smut (Wang et al. 2012; Li et al. 2015), northern corn leaf blight (Ding et al. 2015), southern corn leaf blight (Kump et al. 2011), maize lethal necrosis (Gowda et al. 2015) and tar spot complex (Cao et al. 2017). Association mapping has shown great potential, but the detection power is fairly low and the method is prone to the discovery of false-positive QTLs (Cao et al. 2017). Combining the two mapping approaches to identify candidate QTLs for complex diseases is more powerful due to increased statistical power and improved mapping resolution. This combined approach has been applied to study the genetic architecture of complex traits, including several diseases of maize, such as gray leaf spot (Mammadov et al. 2015), head smut (Li et al. 2015) and tar spot complex (Mahuku et al. 2016).

Genomic prediction (GP) has the capacity to improve breeding efficiency and increase the rates of genetic gains of the quantitative traits (Crossa et al. 2013; Beyene et al. 2015). GP uses markers that cover the whole genome to predict the breeding values of individuals by capturing the effect of both major and minor genes. In GP, the effect of all markers is estimated simultaneously from a training population that has been both phenotyped and genotyped. A model training population is used to calibrate the prediction model, and selections are made based on these predictions. Using this model, genomic breeding values are computed as the sum of marker effects for untested genotyped lines (Meuwissen et al. 2001). GP of complex diseases like Northern corn leaf blight resistance (Technow et al. 2013), MLN (Gowda et al. 2015) and tar spot (Cao et al. 2017) clearly demonstrated its potential in improving quantitative disease resistance. Thus, linkage mapping, association mapping in segregating populations, and GWAS, combined with an extensive array of genomic resources and genotyping technologies, have increased the power and accuracy to dissect complex traits and identify alleles associated with QTLs for important traits (Ingvarsson and Street 2011). In the present study, we combined linkage mapping with three doubled-haploid (DH) populations and GWAS in a global collection of 380 diverse tropical/subtropical maize inbred lines in conjunction with GP using genotyping-by-sequencing (GBS) SNPs. The objectives of this study were (1) to evaluate a diverse array of tropical and subtropical maize lines and DH populations for their responses to MCMV and MLN under artificial inoculation; (2) to conduct individual population-based QTL mapping and joint linkage association mapping (JLAM) to dissect the genetic architecture of MCMV and MLN resistance; (3) to validate the genomic regions through GWAS; and (iv) to assess the potential of GP for MCMV and MLN resistance in maize.

## Materials and methods

Three DH populations (DH pop1-CML550xCML504, 219 lines; DH pop2-CML550xCML511, 110 lines; DH pop3-CML550xCML494, 229 lines) developed from four parents were used for linkage mapping and JLAM. In addition, one association mapping panel—the IMAS (improved maize for African soil; Wen et al. 2011; Gowda et al. 2015) panel comprising 380 inbred lines constituted by the International Maize and Wheat Improvement Center (CIMMYT) was used to evaluate the genetic architecture of MCMV and MLN resistance.

DH lines from three different populations and the IMAS association panel were evaluated for MCMV in a large screenhouse to avoid any mixing with other viruses and for MLN in a quarantined field site using artificial inoculation, undertaken at the MLN Screening Facility at the Kenya Agriculture and Livestock Research Organization (KALRO, https://mln.cimmyt.org) Research Center at Naivasha (latitude 0°43′S, longitude 36°26′E, 1896 m asl), Kenya. All the trials were evaluated for three seasons between 2014 and 2016, except the IMAS panel under MLN, which was evaluated in 2013 and 2014. For all the trials, each experimental unit consisted of 3-m-long single-row plots arranged in an α-lattice design with two replications. To ensure a uniform number of plants per germplasm entry, two seeds were planted per hill and thinned to a single plant per hill 3 weeks after emergence. Standard agronomic practices were followed for each trial.

### Viral inoculum, artificial inoculation and phenotyping

The SCMV and MCMV isolates used in this study for artificial inoculation of the germplasm entries with MLN were initially collected and isolated from infected maize fields in MLN hotspot areas in Kenya. The amplified isolates used were verified as SCMV and MCMV isolates via an enzyme-linked immunosorbent assay (ELISA). To maintain their purity, both SCMV and MCMV inoculums were maintained on the susceptible maize hybrid H614 under isolated greenhouse conditions at the Naivasha MLN Screening Facility until inoculation of germplasm entries in the MCMV screenhouse and MLN field trials. Plants used for inoculum increase were inoculated at the 4–5-leaf stage, and leaves from inoculated plants were used as an inoculum source. The MCMV inoculum for the screenhouse trials and the MLN inoculum for field trials were prepared by following an optimized protocol (Mahuku et al. 2015; Gowda et al. 2018).

Inoculum for the MLN field trial was prepared by following an optimized combination of the SCMV and MCMV viruses (ratio of 4:1). The infected leaves were weighed, chopped and homogenized in 0.1 M potassium phosphate buffer in a 1:10 dilution at pH 7.0. The inoculum was sieved through a nylon mesh paint strainer and 0.02 g/ml of Celite was added. MCMV inoculum for the screenhouse trials and MLN inoculum for field trials were applied mechanically by using a motorized, backpack mist blower (Solo 423 Mist Blower, 12 L capacity). An open-nozzle (2-in. diameter) was used to deliver inoculum spray at a pressure of 10 kg/cm^2^. Inoculation was done twice in 1-week intervals to ensure uniform inoculation. Across all trials, any symptomatic plants observed before inoculation were discarded. The presence of MCMV alone in the screenhouse trials and both viruses (MCMV and SCMV) in the field trials was confirmed by ELISA. MCMV and MLN disease severity (DS) were visually scored on each plot in an ordinal scale of 1 (highly resistant, with no disease symptoms) to 9 (highly susceptible, leading to necrosis and death). Data were recorded at 10-day intervals, beginning from 10 days after the second inoculation for up to five observations. For the DS analyses, after analyzing each time score, we used a third score (40 days post-inoculation) which also had high heritability compared to other scores. The area under the disease progress curve (AUDPC) was calculated for each plot to provide a measure of the progression of MCMV and MLN severity across time (Jones et al. 2007) by using SAS 9.4 (SAS Institute Inc 2015).

### Phenotypic data analyses

Analysis of variance was conducted for DS (40 dpi) and AUDPC data for MCMV and MLN. Analyses were carried out for each DH population and the IMAS association mapping panel across environments by using the PROC MIXED procedure with the restricted maximum likelihood (REML) option in SAS 9.4 (SAS Institute 2015) with the following statistical model:

$$Y_{ijko} = \mu + G_{i} + L_{j} + \left({GL} \right)_{ij} + R\left(L \right)_{kj} + B\left({R.L} \right)_{ojk} + e_{ijko},$$

where *Y*_*ijko*_ is the phenotypic observation for the *i*th genotype at the *j*th environment in the *o*th incomplete block of the *k*th replication, *μ* is an intercept term, *G*_*i*_ is the genetic effect of the *i*th genotype, *L*_*j*_ is the effect of the *j*th environment, (*GL*)_*ij*_ is the interaction effect between genotype and environment, *R*(*L*)_*kj*_ is the effect of the *k*th replication at the *j*th environment, *B(R.L)*_*ojk*_ is the effect of the *o*th incomplete block in the *k*th replication at the *j*th environment, and *e*_*ijko*_ is the residual. The effect of genotype, genotype X environment interaction and incomplete blocks was treated as random to estimate their variances and the residual error variance.

For each phenotypic observation, a mixed linear model (MLM) was fitted by using MEATA-R software (http://hdl.handle.net/11529/10201) to obtain both the best linear unbiased estimate (BLUE) and the best linear unbiased predictor (BLUP) for each genotype across environments. For JLAM, combined analyses of the three DH populations were carried out to calculate both BLUEs and BLUPs and total variance components by using MEATA-R software (http://hdl.handle.net/11529/1020). Heritability (*h*^2^) for the DS and AUDPC values of MLN and MCMV was estimated on a progeny mean basis as: *H*^*2*^ = *σ*_G_^2^/(*σ*_G_^2^ + *σ*_GXE_^2^/*L* + *σ*_e_^2^/LR), where *σ*_G_^2^, *σ*_GXE_^2^, *σ*_e_^2^ referred to the genotypic, genotype X environment interaction and error variances, and L and R indicated the number of environments and replications, respectively.

### Genotyping and linkage mapping

Four parental lines and their DH progenies, and the inbred lines in the IMAS panel were genotyped with high-density markers using GBS at the Institute for Genomic Diversity, Cornell University, Ithaca, USA, as per the procedure described in earlier studies (Elshire et al. 2011; Glaubitz et al. 2014; Gowda et al. 2015). For the three DH populations, the GBS data were filtered with a minor allele frequency (MAF) of > 0.05 and a minimum count of 90% of the sample size. Further, the number of SNPs in each population was reduced by selecting only homozygous and polymorphic markers between the two parents in each population. Linkage maps in all the three DH populations were constructed using QTL IciMapping, version 4.1 software (http://www.isbreeding.net). Highly correlated SNPs, which cannot provide additional information in each population, were removed by an inbuilt tool called BIN implemented in QTL IciMapping. The remaining high-quality SNP data were used to construct genetic linkage maps using the MAP function (Meng et al. 2015), which uses stepwise regression to select the most significant markers and a likelihood ratio test to calculate the logarithm of odds (LOD) scores for each marker by a criterion of > 3.0 LOD and a maximum distance of 30 cM between two loci. Three steps are involved in building a linkage map: grouping, ordering and rippling. Grouping was done with a LOD score of > 3.0, the REcombination Counting and ORDering (RECORD) algorithm was used for ordering markers, and the Sum of adjacent criterion (SAD) ripple was performed to confirm the marker order. Recombination frequencies between two linked loci were transformed into cM using the Kosambi (1944) mapping function.

For each population, BLUPs across environments for DS and AUDPC values for both MCMV and MLN were used to detect QTLs based on inclusive composite interval mapping (ICIM). For QTL analysis, the probability in the stepwise regression was set at 0.01 and the scanning step was 1 cM. A threshold LOD score of > 3.0 was set by using 1000 permutations and a *P* value ≤ 0.05 to determine QTL significance. The phenotypic variation explained (PVE) by each QTL and across all QTLs for each trait was estimated (Tuberosa et al. 2002). The origin of the favorable allele for MCMV and MLN resistance was identified based on the sign of the additive effects of each QTL. In the QTL naming the letter “*q*” indicates QTL, and the abbreviation of the trait name, the chromosome and the marker position follow this.

### Joint linkage association mapping

Three DH populations that were genotyped with GBS were used for JLAM. For quality screening, SNPs that were either monomorphic between any of the two parental lines, or had missing values of > 5% and a minor allele frequency of < 0.05 were discarded from the analysis. After these quality checks, 8000 high-quality GBS SNPs were retained for further analyses across populations. These high-quality SNPs were used to construct an integrated linkage map where markers are arranged based on their physical position by using IciM mapping, version 4.1 software. BLUPs calculated across populations and environments were used in the JLAM studies. A biometric model, which performs well compared to other models for association mapping in multiple biparental populations (Würschum et al. 2012), was used to conduct the JLAM. This model incorporates population effect to control the differences in population means, cofactors to control the genetic background, and a marker effect across populations (Liu et al. 2011). This model is explained in detail by Liu et al. (2011) and Würschum et al. (2012). In brief, with this model, a two-step procedure was followed to find the QTL. First, there was a selection of cofactors based on the Schwarz Bayesian Criterion (SBC, Schwarz 1978) by including a population effect and cofactors. PROC GLM SELECT implemented in the statistical software SAS 9.4 (SAS Institute Inc 2015) was used to select the cofactors. In the second step, *P* values for the *F*-test were calculated by using a full model (including SNP effect) versus a reduced model (without SNP effect). Genome-wide scans for QTLs were implemented in R version 3.2.5 (R Development Core Team 2015). The model used in the present study was as follows:

$${\mathbf{Y}} = {\mathbf{l}}\mu + {\mathbf{X}}_{{\mathbf{D}}} {\mathbf{M}}_{{\mathbf{D}}} + {\mathbf{X}}_{{\mathbf{q}}} b_{q} + \mathop \sum \limits_{c \ne q} {\mathbf{X}}_{{\mathbf{c}}} b_{c} +\varvec{\varepsilon},$$

where **Y** is a *N* × 1 column vector of the BLUP values of phenotypic data of *N* DH lines (*N *= 558) coming from *D* populations (*D *= 3); **l** is a *N* × 1 column vector containing the constant 1; *μ* is the intercept; ${\mathbf{X}}_{{\mathbf{q}}}$ (${\mathbf{X}}_{{\mathbf{c}}}$) is a *N* × 1 column vector containing the SNP types (delegated by 0-1-2) of each individual at marker *q* (cofactor *c*); $b_{q}$ ($b_{c}$) is the expected substitution effect of marker *q* (cofactor *c*); ${\mathbf{X}}_{{\mathbf{D}}}$ is a *N* × *D* matrix whose elements were 0 or 1 according to whether or not a progeny *i* belonged to population *D*; ${\mathbf{M}}_{{\mathbf{D}}}$ is a *D* × 1 vector of population effects; and **ε** is the vector of the residuals of the model. The Bonferroni–Holm procedure (Holm 1979) was used to detect markers with significant (*P* < 0.05) main effects and was controlled for multiple testing. The total proportion of PVE by the detected QTLs was calculated by fitting all significant SNPs simultaneously in a linear model to obtain an adjusted *R*^2^ (Utz et al. 2000).

### Genome-wide association analyses

The IMAS association mapping panel comprises 380 inbred lines; detailed information of these inbred lines and their genotyping are described in our previous study (Gowda et al. 2015). TASSEL Ver 5.2 (Bradbury et al. 2007) was used to filter raw GBS datasets for SNPs where a minor allele frequency (MAF) of < 0.02, heterozygosity of > 5% and missing data rates > 5% were excluded from further analyses. After these quality checks, 293,106 high-quality SNPs were retained for GWAS. The association panel was planted in screenhouses for three seasons in 2014 and 2015 to screen for MCMV resistance, and the same set of inbred lines were also evaluated for MLN in the field under artificial inoculation in Naivasha for three seasons. Details of the MLN screening and data scoring are explained in our earlier study (Gowda et al. 2015). The BLUP values for DS and AUDPC of MCMV and MLN traits across environments were used as phenotypes for GWAS.

The principal component analysis (PCA) was carried out according to Price et al. (2006), implemented in SNP & Variation Suite (SVS) V_8.6.0 (SVS, Golden Helix, Inc., Bozeman, MT, www. goldenhelix.com). A two-dimensional plot of the first two principal components (PCs) was created to visualize the possible population stratification among the samples (Supplementary Fig S1). The extent of genome-wide LD was based on adjacent pairwise *r*^2^ values between high-quality SNPs from the GBS and physical distances between these SNPs (Remington et al. 2001). Nonlinear models with *r*^2^ as responses (*y*) and pairwise distances (*x*) as predictors were fitted into the genome-wide LD data using the “nlin” function in R (R core team 2015). Average pairwise distances in which LD decayed at *r*^2^ = 0.2 and *r*^2^ = 0.1 were calculated (Hill and Weir 1988). PCA was calculated across all DH population and IMAS association mapping panel by using TASSEL, and the first three PCs were plotted by CurlyWhirly v1.15 (http://ics.hutton.ac.uk/curlywhirly/; Supplementary Fig S3).

For GWAS, a mixed linear model was used where population structure was corrected by using both PCs and kinship (K) (Flint-Garcia et al. 2005; Yu and Buckler 2006). The kinship matrix was calculated with a centered IBS option by using TASSEL ver 5.2 (Bradbury et al. 2007). The first three PCs were used to correct for the population structure. Genome-wide scans for marker–trait associations were conducted to detect main-effect QTLs. The amount of phenotypic variation explained by the model was assessed using R^2^ statistics, calculated by fitting all significant SNPs simultaneously in a linear model. Multiple testing correction was performed to determine the significance threshold, where instead of 293,106 independent tests, the total number of tests were estimated based on the average extent of LD at *r*^2^ = 0.1 (Cui et al. 2016). Based on this, significant associations were declared when the *P* values in independent tests were less than 5.8 × 10^−05^. Candidate genes containing or being adjacent to the significant SNPs were obtained from the B73 gene set in Maize GDB (https://www.maizegdb.org/gene_center/gene).

### Genomic prediction

GP was carried out with ridge-regression BLUP (RR-BLUP) with fivefold cross-validation. BLUEs across location were used for the GP analysis. From the GBS data, a subset of 4000 SNPs distributed uniformly across the genome, with no missing values, and minor allele frequency > 0.05 were used for GP in each DH population and IMAS panel. Details of the implementation of the RR-BLUP model are described by Zhao et al. (2012). Three GP approaches differing in the composition of the training set were evaluated with respect to the prediction accuracy for lines in the testing set: (1) “within-population” prediction, where lines within either the DH population or IMAS panel were sampled to form both a training set and testing set; (2) “combined-population” prediction, where all populations are combined and randomly sampled to form both a training set and testing set; and (3) “across-population” prediction, where a training set is sampled from one population and a testing population is sampled from other population; here, the IMAS association mapping panel was used as a training set and each of the DH populations was used as a different testing set. The prediction accuracy was calculated as the correlation between genomic estimated breeding values (GEBVs) and the observed phenotypes divided by the square root of the heritability estimated in the respective populations (Dekkers 2007). Sampling of the training and validation sets was repeated 100 times for each approach.

## Results

**Fig. 1:**
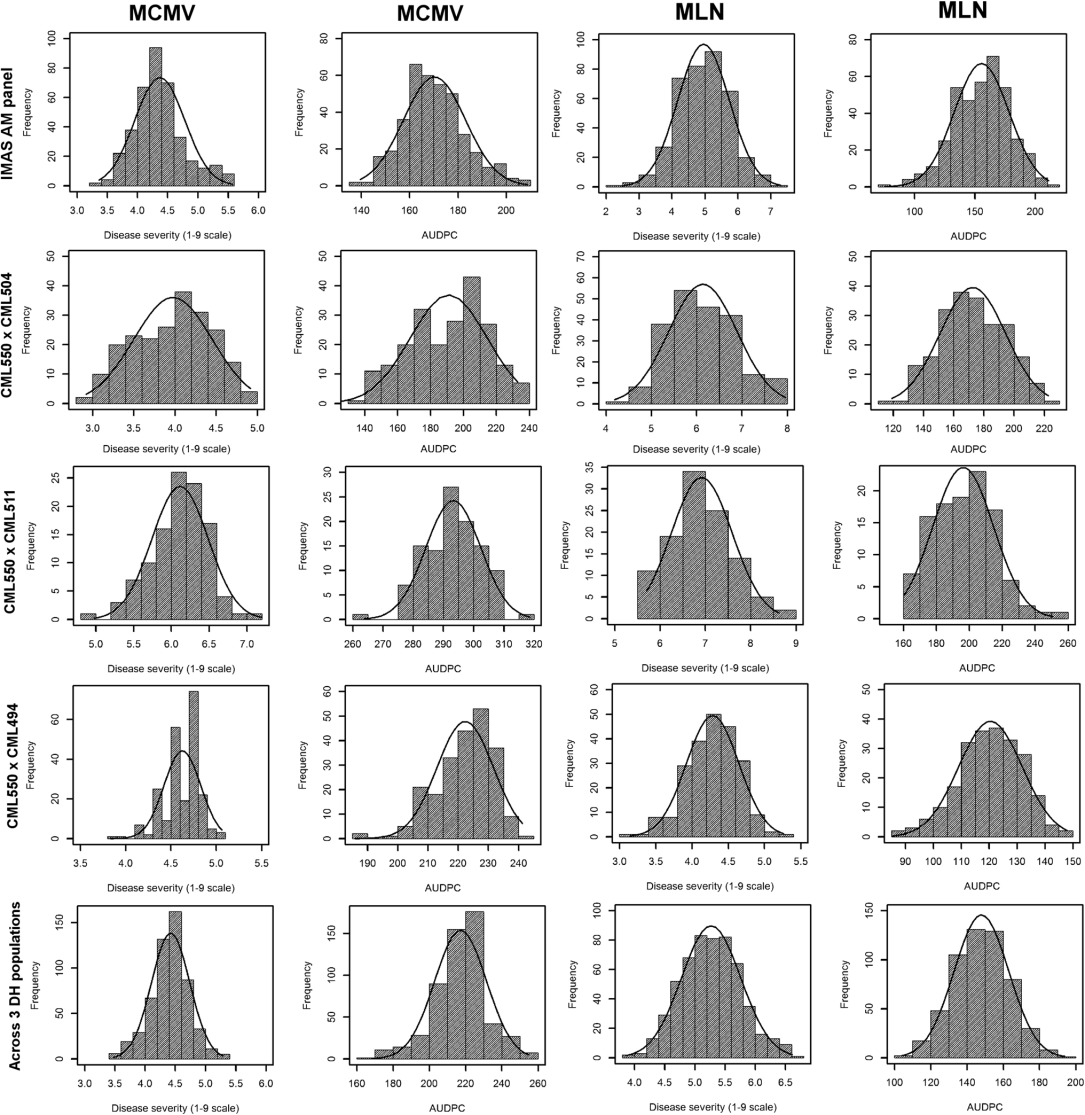
Phenotypic distribution of disease severity and the AUDPC values for MCMV and MLN on a 1–9 scale in three DH populations, combined DH populations, and the IMAS panel

A considerable variation was observed in the DS and AUDPC values of MCMV and MLN in all three DH populations and in the IMAS panel (Fig. 1, Table 1). Among the four CMLs used as parents of the DH populations, CML550 and CML494 had mean scores of 3.5 and 4.0 for the DS values of MCMV (MCMV-DS) and 4 and 5 for MLN (MLN-DS), respectively. In contrast, CML504 was moderately tolerant with mean scores of 4.5 and 6 for MCMV-DS and MLN-DS, respectively. CML511 was susceptible with mean scores of 6 and 8 for MCMV-DS and MLN-DS, respectively. We observed a wide variation in both MCMV-DS and MLN-DS, as well as the respective AUDPC values (Fig. 1). The phenotypic means ranged from 2.92 to 7.19 for MCMV-DS, and from 3.15 to 8.61 for MLN-DS among the three DH populations. Combined analyses of the three DH populations revealed an average DS of 4.42 and 5.28 for MCMV and MLN, respectively. The IMAS association mapping panel showed a range of 3.30–5.60 for MCMV-DS and 2.48–7.29 for MLN-DS (Table 1).

**Table 1:** Means, ranges and components of variance for DS and area under disease progress curve (AUDPC) for maize inbred lines from IMAS association panel and three DH populations inoculated with MCMV and MLN viruses

Trait	Mean (range)	*σ*_G_^2^	*σ*_GE_^2^	*σ*_e_^2^	*h* ^2^
CML550 X CML504 (DH pop1)					
MCMV-DS	3.98 (2.92–4.92)	0.31**	0.13**	0.39	0.74
MCMV-AUDPC	191.10 (136.00–232.90)	726.36**	379.76**	541.94	0.77
MLN-DS	6.14 (4.19–7.96)	0.77**	0.00	0.39	0.80
MLN-AUDPC	172.6 (118.90–222.70)	598.53**	0.00	188.41	0.86
CML550 X CML511 (DH pop2)					
MCMV-DS	6.11 (4.97–7.19)	0.10*	0.04**	0.23	0.57
MCMV-AUDPC	293.20 (263.9–318.5)	59.84*	42.26*	436.74	0.31
MLN-DS	6.91 (5.70–8.61)	0.62**	0.00	0.31	0.80
MLN-AUDPC	196.30 (160.60–250.90)	453.92**	0.00	202.81	0.82
CML550 X CML494 (DH pop3)					
MCMV-DS	4.60 (3.83–5.20)	0.09**	0.00	0.24	0.43
MCMV-AUDPC	222.48 (185.90–241.30)	197.71**	0.00	323.93	0.55
MLN-DS	4.50 (3.15–5.30)	0.18**	0.08**	0.32	0.73
MLN-AUDPC	120.78 (85.33–147.98)	164.63**	39.66**	198.38	0.81
Combined DH populations					
MCMV-DS	4.42 (3.47–5.33)	0.21**	0.11**	0.36	0.78
MCMV-AUDPC	217.20 (168.40–256.40)	379.82**	274.28**	476.56	0.82
MLN-DS	5.28 (3.85–7.18)	0.36**	0.09**	0.34	0.89
MLN-AUDPC	148.24 (103.79–202.95)	312.48**	45.27**	199.13	0.92
IMAS AM panel					
MCMV-DS	4.37 (3.30–5.60)	0.34**	0.39**	0.80	0.56
MCMV-AUDPC	170.43 (139.50–208.80)	310.57**	352.30**	562.51	0.60
MLN-DS	4.96 (2.48–7.29)	0.94**	0.31**	1.66	0.71
MLN-AUDPC	155.73 (40.37–240.81)	803.27**	294.99**	1180.48	0.73

*, **Significance at *P *< 0.05 and *P *< 0.01, respectively

Consistent with the phenotypic observations, ANOVA across environments revealed significant genotypic variances for both MCMV-DS and MLN-DS, as well as for the AUDPC values in each DH population and across DH populations and also for the IMAS panel (Table 1). For MCMV-DS, the GxE interaction variance was significant in all populations except for DH pop3, whereas for MLN-DS, the GxE interaction variance was significant only for DH pop3, across DH populations and the IMAS panel. Heritability (*h*^2^) estimates ranged from moderate to high with 0.43 in DH pop3 to 0.78 across the DH populations for MCMV-DS, and from 0.31 in DH pop2 to 0.82 across the DH populations for MCMV-AUDPC. The estimates of heritability for MLN-DS and MLN-AUDPC ranged from 0.71 in the IMAS panel to 0.89 across the DH populations and 0.73 in IMAS panel to 0.92 across the DH populations, respectively (Table 1). For each population, there was adequate expression of the disease to differentiate tolerant and susceptible lines in each environment. From phenotypic evaluation of lines for MCMV and MLN responses, 12 lines that had a resistance response in all the environments were identified as the best (Supplementary Table S1).

Linkage maps were constructed for all three DH populations. The number of progenies, SNPs, map lengths, and average genetic distances between SNPs for each population are presented in Supplementary Table S2. For MCMV-DS, in DH pop1 a set of five QTLs were detected, which individually explained 1.9–49.9% of the phenotypic variance and together explained 67.9% of the total phenotypic variance. In DH pop2 and DH pop3, two and three QTLs were detected for MCMV-DS, respectively. These QTLs individually explained 11.1–15.7% and 5.2–30.6% of the phenotypic variance and together explained 28.8 and 12.5% of the total phenotypic variance in DH pop2 and DH pop3, respectively (Table 2). For MCMV-AUDPC, we identified a set of five, one and three QTLs in DH pop1, DH pop2 and DH pop3, respectively. The phenotypic variances explained by these QTLs ranged from 1.9 to 58.7% in DH pop1 and 6.7 to 30.5% in DH pop3. The total PVE explained by these QTLs for AUDPC was 72.8%, 10.2% and 13.1% in DH pop1, DH pop2 and DH pop3, respectively. The QTLs for MCMV resistance were found on maize chromosomes 1, 2, 3, 4, 5, 7, 8 and 9 (Table 2). One QTL detected on chromosome 3, *qMCMV3*-*108*, explained > 53% of the total phenotypic variation, and was found to have the largest effect.

**Table 2:** Detection of QTL associated with resistance to MCMV and MLN (based on disease severity and AUDPC value), their physical positions and genetic effects in three DH populations

Virus	Trait	QTL name	Chr	Position (cM)	LOD	PVE (%)	Add	Total PVE (%)	QTL confidence interval		Favorable allele from
									Left M	Right M	
CML550 X CML504 DH pop											
MCMV	Disease severity	*qMCMV1*-*80*	1	191	6.24	4.65	− 0.10	67.89	S1_80964123	S1_84413650	CML504
		*qMCMV2*-*96*	2	98	4.16	2.92	− 0.08		S2_95377918	S2_96811777	CML504
		*qMCMV3*-*108*	3	39	43.97	49.87	0.34		**S3_109388419**	**S3_108706910**	CML550
		*qMCMV5*-*147*	5	51	2.76	1.93	0.07		S5_131040959	S5_147478496	CML550
		*qMCMV9*-*136*	9	82	3.89	2.72	− 0.08		S9_136073220	S9_133546700	CML504
	AUDPC	*qMCMV1*-*10*	1	88	3.54	4.76	− 5.11	72.80	S1_191199656	S1_8038925	CML504
		*qMCMV1*-*76*	1	185	3.28	1.96	− 3.25		S1_75202736	S1_76633047	CML504
		*qMCMV2*-*148*	2	89	4.27	2.57	− 3.73		S2_148564919	S2_144479730	CML504
		*qMCMV3*-*108*	3	39	53.91	58.70	17.81		**S3_109388419**	**S3_108706910**	CML550
		*qMCMV8*-*169*	8	105	4.17	4.22	− 4.78		S8_149983936	S8_169645402	CML504
MLN	Disease severity	*qMLN3*-*108*	3	38	20.81	21.43	0.35	60.63	**S3_85659716**	**S3_109388419**	CML550
		*qMLN3*-*167*	3	72	11.23	15.24	0.29		**S3_167432530**	**S3_25921599**	CML550
		*qMLN4*-*123*	4	105	3.88	3.34	− 0.14		S4_123186378	S4_96312333	CML504
		*qMLN6*-*158*	6	183	3.67	3.53	0.14		**S6_168794605**	**S6_158282777**	CML550
	AUDPC	*qMLN3*-*108*	3	38	25.34	23.73	10.56	65.92	**S3_85659716**	**S3_109388419**	CML550
		*qMLN3*-*167*	3	72	9.46	11.02	7.21		**S3_167432530**	**S3_25921599**	CML550
		*qMLN4*-*123*	4	105	4.15	3.06	− 3.80		S4_123186378	S4_96312333	CML504
		*qMLN6*-*158*	6	188	3.75	2.78	3.62		**S6_158282777**	**S6_142569154**	CML550
		*qMLN7*-*140*	7	61	3.08	2.29	− 3.28		S7_149357653	S7_139724362	CML504
		*qMLN7*-*02*	7	273	2.63	4.25	− 4.47		S7_995619	S7_2133516	CML504
CML550 X CML511 DH pop											
MCMV	Disease severity	*qMCMV2*-*192*	2	205	3.44	11.14	0.05	28.81	**S2_186533942**	**S2_192598964**	CML550
		*qMCMV3*-*108*	3	229	4.87	15.70	0.07		**S3_113425715**	**S3_51499448**	CML550
	AUDPC	*qMCMV3*-*108*	3	223	2.75	10.86	2.99	10.29	**S3_119322983**	**S3_85659716**	CML550
MLN	Disease severity	*qMLN3*-*108*	3	228	9.74	33.00	0.19	33.15	**S3_66944571**	**S3_113425715**	CML550
	AUDPC	*qMLN3*-*108*	3	223	7.50	26.62	9.66	26.21	S3_119322983	S3_85659716	CML550
CML550 X CML494 DH pop											
MCMV	Disease severity	*qMCMV1*-*290*	1	123	4.67	9.56	− 0.06	12.45	S1_296767813	S1_290856560	CML494
		*qMCMV4*-*235*	4	10	3.91	30.61	− 0.16		**S4_28982373**	**S4_235268661**	CML494
		*qMCMV7*-*132*	7	154	2.80	5.20	− 0.04		S7_132165418	S7_135891516	CML494
	AUDPC	*qMCMV1*-*290*	1	123	4.73	9.72	− 1.65	13.07	S1_296767813	S1_290856560	CML494
		*qMCMV4*-*235*	4	22	2.66	30.56	− 3.50		**S4_28982373**	**S4_235268661**	CML494
		*qMCMV7*-*132*	7	137	3.60	6.71	− 1.36		S7_129265174	S7_132165418	CML494
MLN	Disease severity	*qMLN4*-*235*	4	88	3.64	6.55	− 0.09	25.43	S4_235268661	S4_230348667	CML494
		*qMLN5*-*135*	5	200	2.75	4.06	− 0.12		S5_85865774	S5_135455214	CML494
		*qMLN6*-*89*	6	213	6.13	9.29	− 0.11		S6_93323515	S6_89823772	CML494
		*qMLN7*-*152*	7	191	5.99	12.88	− 0.13		**S7_152064248**	**S7_166556623**	CML494
	AUDPC	*qMLN3*-*29*	3	169	2.81	3.75	2.25	31.99	S3_28920381	S3_35353666	CML550
		*qMLN4*-*235*	4	88	6.46	10.66	− 3.73		**S4_235268661**	**S4_230348667**	CML494
		*qMLN4*-*15*	4	200	3.34	4.65	− 2.49		S4_20053786	S4_14632354	CML494
		*qMLN6*-*89*	6	214	4.82	6.70	− 2.95		**S6_89823772**	**S6_87406549**	CML494
		*qMLN7*-*152*	7	186	7.16	13.57	− 4.21		**S7_152064248**	**S7_166556623**	CML494

Markers with bold letters are the QTL consistent across DS and AUDPC values

*LOD* logarithm of odds; *Add* additive effect; *PVE* phenotypic variance explained

For MLN, we found a set of four, one and four QTLs for DS and six, one and five QTLs for AUDPC for DH pop1, DH pop2 and DH pop3, respectively (Table 2). Among these DH populations, the phenotypic variance explained by individual QTLs for MLN-DS ranged from 3.3 to 33%, whereas the range was 2.2–26% for the AUDPC values. The highest total phenotypic variance explained was 65.9% for MLN-AUDPC in DH pop1 and the lowest was 25.4% for MLN-DS in DH pop3 (Table 2). The QTL detected on chromosome 3, *qMCMV3*-*108*, which explained 33% of the total phenotypic variation, was found to have the largest effect followed by same QTL for AUDPC, which explained 26.6% of the total phenotypic variation. Interestingly, the favorable alleles for both QTLs were derived from CML550, which was the MCMV- and MLN-tolerant parent.

**Fig. 2:**
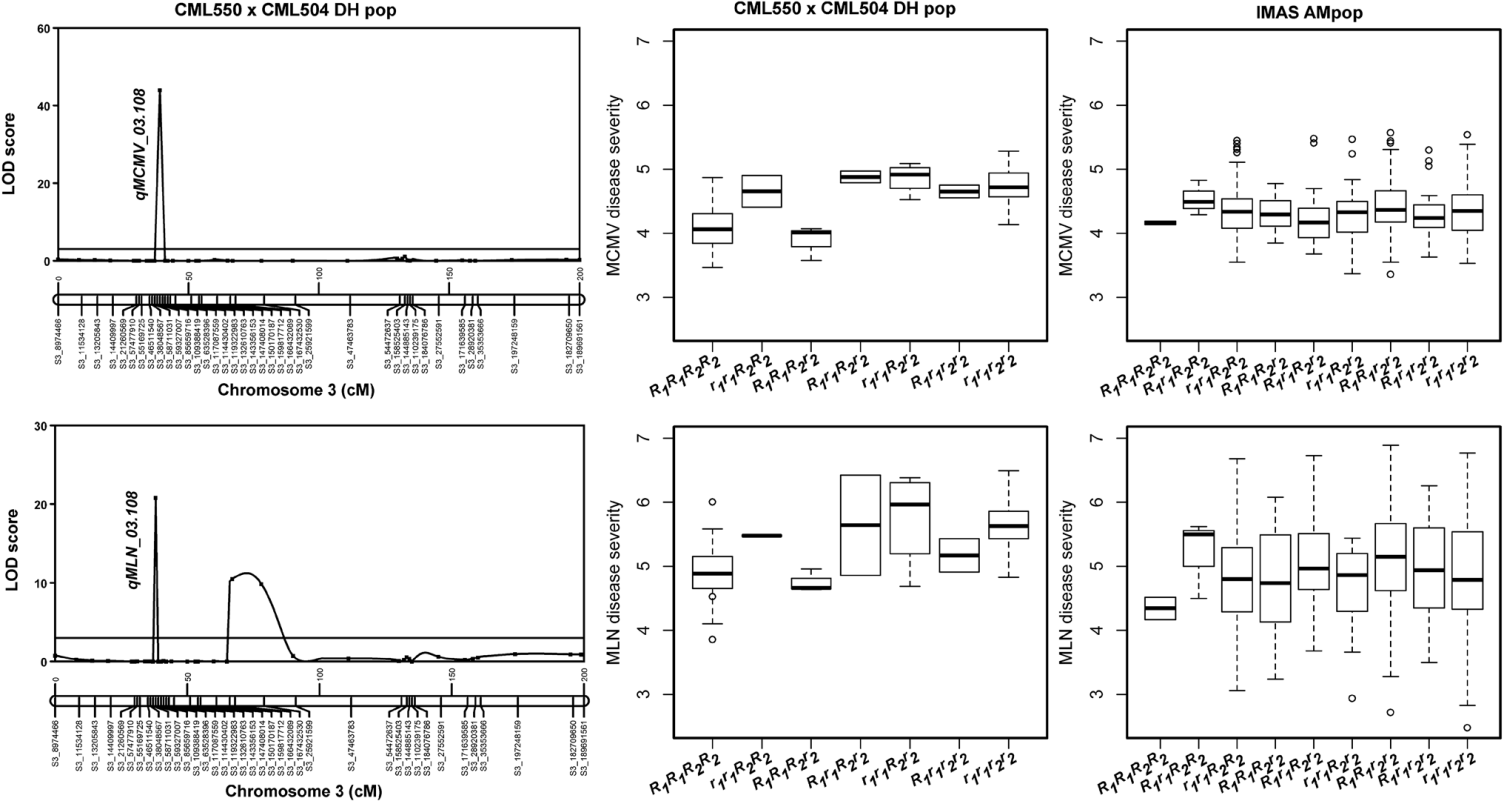
Major QTL for MCMV and MLN resistance in the DH populations. A likelihood of odds (LOD) scan showing the QTLs identified on chromosome 3. Box–whisker plots display the level of disease resistance or severity for different allele combinations at resistance gene loci explaining > 20% of the phenotypic variation for MCMV and MLN as determined by two strongly associated SNP markers

Combined analyses of DH populations through JLAM revealed 10 QTLs each for MCMV-DS and AUDPC values, which distributed across all chromosomes except chromosome 10 (Table 3). These QTLs individually explained 0.4–41% of the phenotypic variance for DS and 1.1–29.4% of the phenotypic variance for AUDPC values. Two QTLs (*qMCMV2*-*189* and *qMCMV6*-*17*) were common to both the DS and AUDPC values, while the others were specific. QTL *qMCMV3*-*108* detected on chromosome 3 was the largest effect QTL, and it explained 41.6% of phenotypic variance followed by *qMCMV6*-*17* on chromosome 6 which explained 29.4% of phenotypic variance. QTL *qMCMV6*-*17* was consistently detected for both DS and AUDPC values. For MCMV, all the detected QTLs together explained 58% and 67% of total phenotypic variance for DS and AUDPC, respectively. JLAM analyses for MLN revealed nine QTLs associated with DS and 14 QTLs associated with AUDPC values (Table 3). Two QTLs (*qMLN6*-*17* and *qMLN7*-*144*) were common for DS and AUDPC values. The PVE explained by individual QTLs for DS ranged from 1.5 to 17.6% and for AUDPC the PVE ranged from 0.9 to 22.9%. For both MLN-DS and MLN-AUDPC, QTL *qMLN6*-*17* was the largest effect QTL, with 17.6% and 22.9% of the PVE, respectively. For MLN-DS, QTL *qMLN3*-*119* was the second largest effect QTL with 10.9% of PVE, and for MLN-AUDPC, *qMLN3*-*87* was the second largest effect QTL with 10.7% of PVE. The total PVE by all the detected QTLs was 50% and 54% for DS and AUDPC, respectively. A major QTL, *qMCMV*-*108/qMLN3*-*108*, identified in DH pop1 indicated that CML 550 is a source of favorable alleles (Fig. 2).

**Table 3:** Analysis of trait-associated markers, allele substitution (*α*) effects, and the total phenotypic variance (*R*^2^) of the joint linkage association mapping based on combined three DH populations

MCMV	QTL name	Chr	Position (Mbp)	Disease severity				AUDPC	
				*α*-effect	*P* value	PVE (%)	*α*-effect	*P* value	PVE (%)
S1_10960822	*qMCMV1*-*10*	1	10.961	–	–	–	− 6.21	3.01E − 24	9.60
S1_71020191	*qMCMV1*-*71*	1	71.020	− 0.10	2.23E − 11	4.40	–	–	–
S2_38977357	*qMCMV2*-*39*	2	38.977	–	–	–	− 3.57	2.07E − 09	3.10
S2_111135899	*qMCMV2*-*111*	2	111.136	− 0.08	1.62E − 08	3.10	–	–	–
S2_189579989	*qMCMV2*-*189*	2	189.580	0.04	3.81E − 02	0.40	11.95	3.04E − 45	20.30
S3_108706910	*qMCMV3*-*108*	3	108.707	0.31	7.87E − 73	41.60	–	–	–
S3_116124132	*qMCMV3*-*116*	3	116.124	0.06	1.62E − 03	0.90	–	–	–
S3_149234811	*qMCMV3*-*149*	3	149.235	–	–	–	2.25	3.39E − 04	1.10
S3_196142479	*qMCMV3*-*196*	3	196.142	− 0.05	4.82E − 04	1.10	–	–	–
S4_163779660	*qMCMV4*-*163*	4	163.780	–	–	–	− 1.27	2.97E − 03	0.80
S5_133915065	*qMCMV5*-*133*	5	133.915	–	–	–	2.41	1.48E − 03	1.20
S5_210676383	*qMCMV5*-*210*	5	210.676	− 0.05	3.20E − 03	1.10	–	–	–
S5_213038590	*qMCMV5*-*213*	5	213.039	0.06	6.34E − 05	2.00	–	–	–
S6_1540161	*qMCMV6*-*2*	6	1.540	–	–	–	− 5.27	1.03E − 08	3.90
**S6_17165743**	*qMCMV6*-*17*	6	17.166	0.19	7.73E − 42	27.20	9.49	3.63E − 47	29.40
S7_5784540	*qMCMV7*-*6*	7	5.785	–	–	–	− 2.16	1.73E − 05	2.20
S8_170127444	*qMCMV8*-*170*	8	170.127	− 0.10	1.93E − 14	7.70	–	–	–
S9_41517817	*qMCMV9*-*41*	9	41.518	–	–	–	− 5.78	1.46E − 15	7.80
Total PVE (%)						0.58			0.67

MLN				Disease severity				AUDPC	
S1_100824500	*qMLN1*-*100*	1	100.825	− 0.27	4.02E − 04	1.50	–	–	–
S1_146484798	*qMLN1*-*146*	1	146.485	0.39	1.76E − 07	3.20	–	–	–
S3_47463783	*qMLN3*-*47*	3	47.464	0.09	8.18E − 05	1.80	–	–	–
S3_86873766	*qMLN3*-*86*	3	86.874	–	–	–	6.92	9.87E − 20	10.70
S3_87781149	*qMLN3*-*87*	3	87.781	–	–	–	− 3.20	8.36E − 04	1.30
S3_119614021	*qMLN3*-*119*	3	119.614	0.22	1.07E − 20	10.90	–	–	–
S3_154250438	*qMLN3*-*154*	3	154.250	0.14	4.02E − 11	5.30	–	–	–
S3_167432530	*qMLN3*-*167*	3	167.433	–	–	–	4.31	1.98E − 07	3.30
S4_90676084	*qMLN4*-*90*	4	90.676	− 0.08	5.09E − 06	2.50	–	–	–
S4_121562618	*qMLN4*-*121*	4	121.563	–	–	–	− 4.51	4.99E − 10	4.80
S4_235268672	*qMLN4*-*235*	4	235.269	–	–	–	− 2.09	2.24E − 04	1.70
S5_201226926	*qMLN5*-*201*	5	201.227	–	–	–	4.28	3.26E − 08	3.30
**S6_17165743**	*qMLN6*-*17*	6	17.166	0.23	3.24E − 27	17.60	8.82	6.16E − 42	22.90
S6_159257330	*qMLN6*-*159*	6	159.257	0.06	5.10E − 04	1.70	–	–	–
S6_164557883	*qMLN6*-*165*	6	164.558	–	–	–	5.37	5.16E − 06	2.20
S7_144659968	*qMLN7*-*144*	7	144.660	− 0.11	1.35E − 08	4.50	− 3.76	3.23E − 13	5.80
S8_22170669	*qMLN8*-*22*	8	22.171	–	–	–	− 2.37	1.76E − 04	1.50
S9_55511793	*qMLN9*-*55*	9	55.512	–	–	–	− 3.19	4.79E − 09	3.70
S10_137828847	*qMLN10*-*137*	10	137.829	–	–	–	− 5.76	3.63E − 09	3.80
S10_147141046	*qMLN10*-*147*	10	147.141	–	–	–	− 3.01	4.34E − 03	0.90
S10_149558048	*qMLN10*-*149*	10	149.558	–	–	–	5.28	2.28E − 07	2.90
Total PVE (%)						0.50			0.54

*Chr Chromosome, *PVE* proportion of phenotypic variance explained

PCA of the IMAS association panel revealed a moderate population structure (Supplementary Fig S1). The first two eigenvectors clearly delineated three clusters comprising lowland tropical lines, subtropical lines and lines from the ARC-South Africa breeding program. The first two PCs explained 15.4% and 8.8% of variation. The genome-wide LD decay plotted as LD (*r*^2^) between adjacent pairs of markers versus distance in kb showed that the average LD decay was 18.82 Kb at *r*^2^ = 0.1 and 6.53 kb at *r*^2^ = 0.2 (Supplementary Fig S2).

**Fig. 3:**
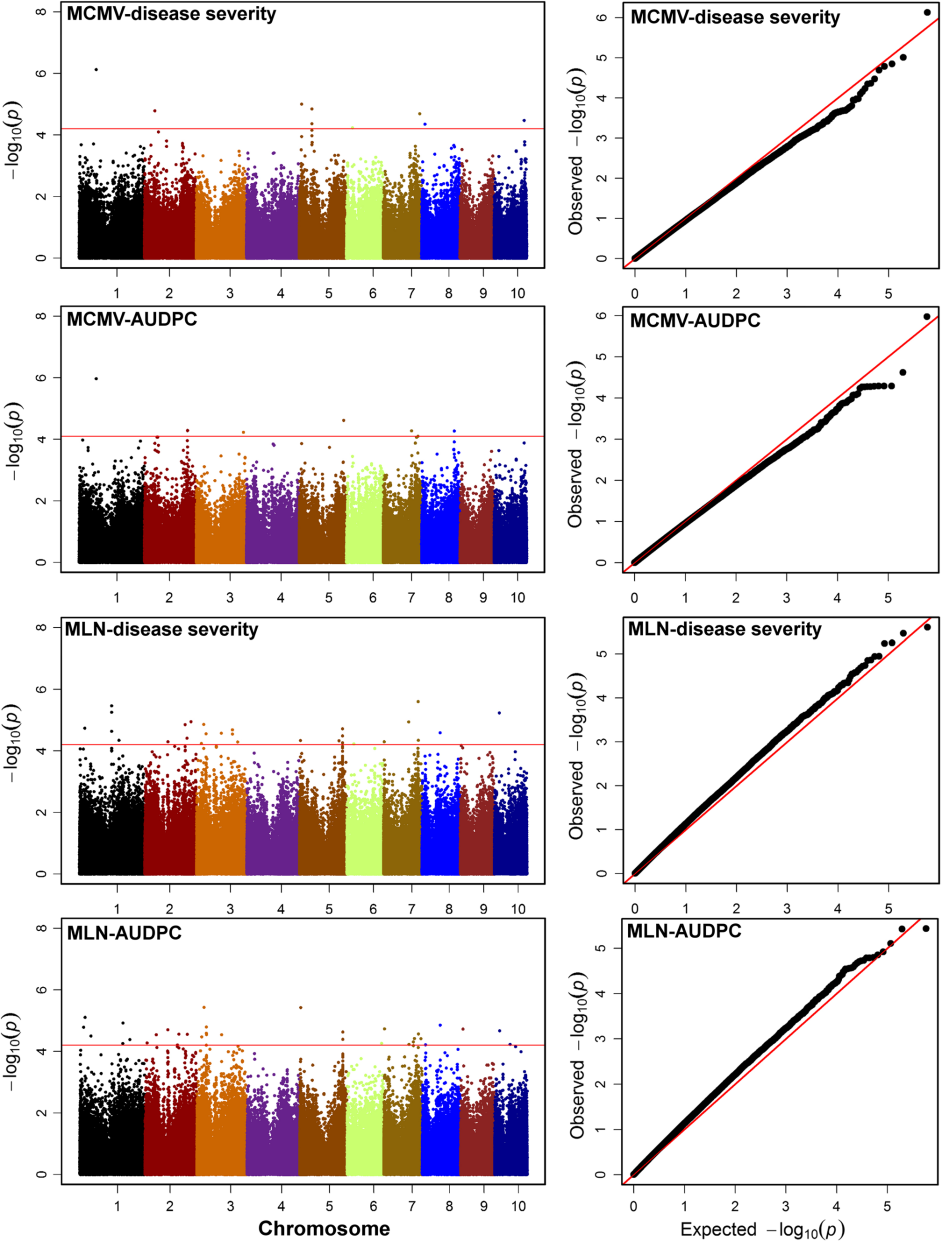
Manhattan plot and *Q*–*Q* plots for the GWAS of MCMV and MLN for disease severity and the AUDPC value in the IMAS association mapping panel. The dashed horizontal line in Manhattan plots depicts the significance threshold (*P* = 5.8 × 10^−5^). The X-axis indicates the SNP location along the 10 chromosomes, separated by different colors; Y-axis is the − log10 (*P* observed) for each analysis. *Q*–*Q* plots depicts inflation of observed versus expected − log10 (*P* values) plots for each trait

In a previous study, we used the IMAS panel to identify the genetic architecture and putative candidate genes underlying MLN resistance by using only the MLN-DS score (Gowda et al. 2015). In this study, we used the same panel to identify and validate the genomic regions for MCMV-DS and MCMV-AUDPC, and MLN-AUDPC. The GWAS results for the DS and AUDPC of both MCMV and MLN are presented as Manhattan plots (Fig. 3). Quantile–quantile plots of P values comparing the expected − log_10_ p value to the observed − log_10_ p value are also shown in Fig. 3. For MCMV, we detected eight and six significant marker–trait associations for DS and AUDPC, respectively (Table 4). These significantly associated SNPs individually explained 5–8% of the total phenotypic variance. Among these significantly associated SNPs, *S1_79444916* on chromosome 1 was found to be the most significantly associated SNP for both DS and AUDPC, which explained 8% of the phenotypic variance.

**Table 4:** Chromosomal position and SNPs significantly associated with MCMV disease severity (DS) and area under disease progress curve (AUDPC) detected by SNP-based GWAS in the IMAS association mapping panel

Trait	SNP-name	Chr	Position (bp)	MCMV	MAF	Minor Allele	Putative candidate gene	Predicted function of candidate gene
			*P* values	*R* ^2^				
Disease severity								
S1_79444916	1	79444916	7.44E − 07	0.08	0.02	C/T	GRMZM2G396640	Uncharacterized protein
S2_47111414	2	47111414	1.63E − 05	0.06	0.02	A/C	GRMZM2G086971	GTPase-mediated signal transduction
S5_11490669	5	11490669	9.81E − 06	0.07	0.48	C/T	GRMZM2G177934	Copper ion binding
S5_58728012	5	58728012	1.41E − 05	0.06	0.03	A/G	GRMZM2G098793	Glycosyltransferase
S6_28146715	6	28146715	5.79E − 05	0.06	0.24	G/A	GRMZM2G313448	Uncharacterized protein
S7_167346730	7	167346730	2.03E − 05	0.06	0.02	A/G	GRMZM2G158130	Uncharacterized protein
S8_14796196	8	14796196	4.46E − 05	0.06	0.03	A/C	GRMZM2G139600	Gamma-glutamyltransferase activity
S10_139328331	10	139328331	3.37E − 05	0.06	0.02	T/C	GRMZM2G125585	Unknown
Total R2				0.23				
AUDPC value								
S1_79444916	1	79444916	1.07E − 06	0.08	0.02	C/T	GRMZM2G396640	Uncharacterized protein
S2_197143379	2	197143379	5.12E − 05	0.06	0.03	G/A	GRMZM2G151656	SAUR52-auxin-responsive SAUR family
S3_217571950	3	217571950	5.84E − 05	0.06	0.10	A/C	GRMZM2G480687	Response to freezing; G protein-coupled receptor protein signaling pathway
S5_205155934	5	205155934	2.42E − 05	0.06	0.06	T/G	GRMZM2G090609	Caleosin-related protein
S7_130133358	7	130133358	5.31E − 05	0.05	0.07	T/C	AC210027.3_FG003	Unknown
S8_149982735	8	149982735	5.39E − 05	0.06	0.47	T/G	GRMZM2G160990	G protein-coupled receptor protein signaling pathway
Total R2				0.21				

For MLN, a set of 20 significant SNPs distributed across six chromosomes were identified for DS that individually explained 5–7% of the total phenotypic variance (Supplementary Table S3), whereas for AUDPC we detected 26 SNPs significantly associated with MLN-AUDPC values, explaining 5–7% of phenotypic variance. *S5_5205032* on chromosome 5 was found to be the most significantly associated SNP for both MLN-DS and MLN-AUDPC. A set of putative candidate genes were identified; based on their functions, these can be grouped as either R genes or plant defense responsive genes (Table 4, Supplementary Table S3). All the QTLs detected for MCMV and MLN in each DH population and JLAM, quantitative trait nucleotides (QTNs) for GWAS were mapped on one integrated physical map (Supplementary Fig S4).

**Fig. 4:**
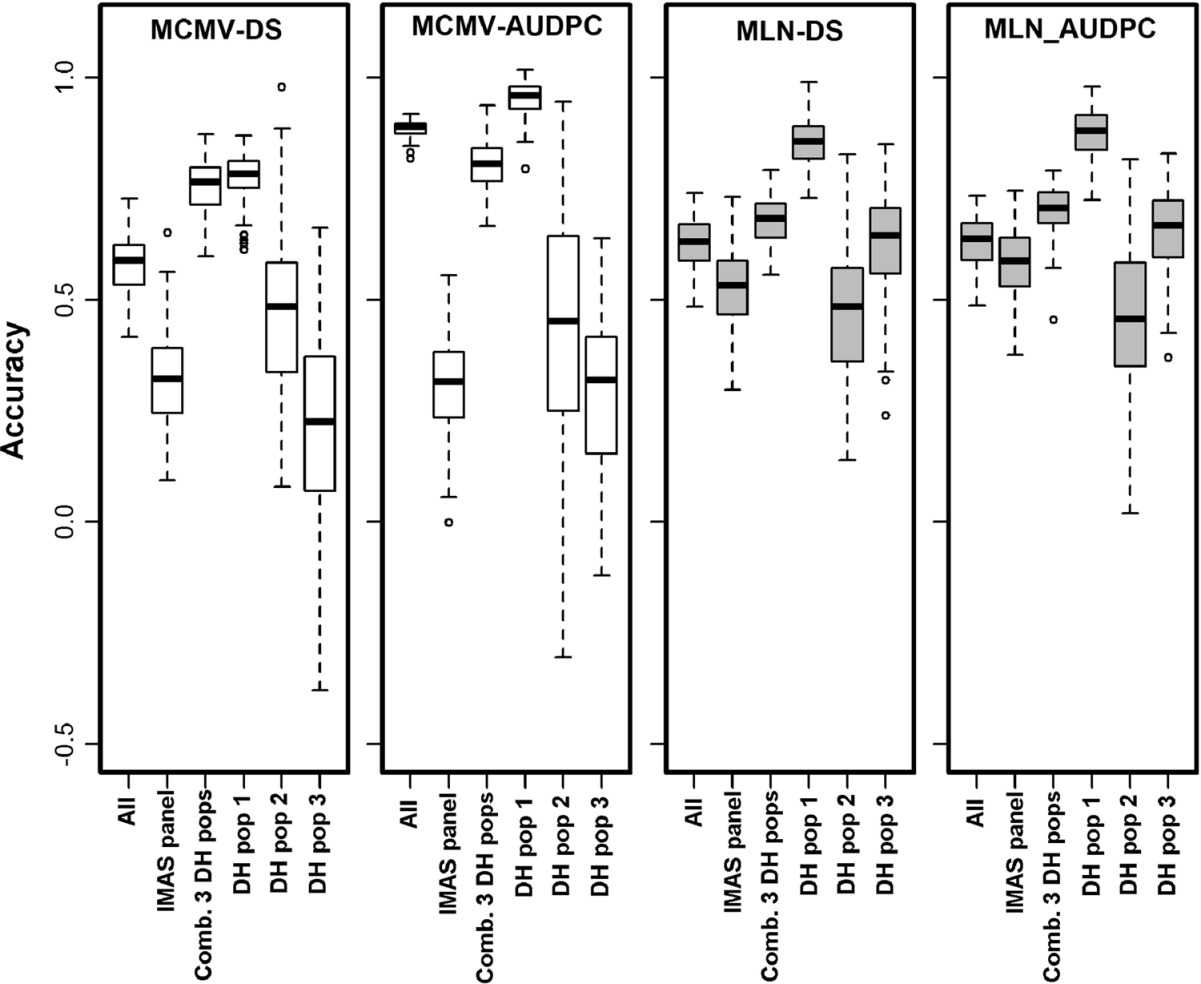
Box–whisker plots for the accuracy of genomic predictions assessed by fivefold cross-validation. Results are shown for the combined association panel and DH populations (all), the IMAS association panel, the combined DH populations and the three individual DH populations for the MCMV-DS, MCMV-AUDPC, MLN-DS, and MLN-AUDPC scores

**Fig. 5:**
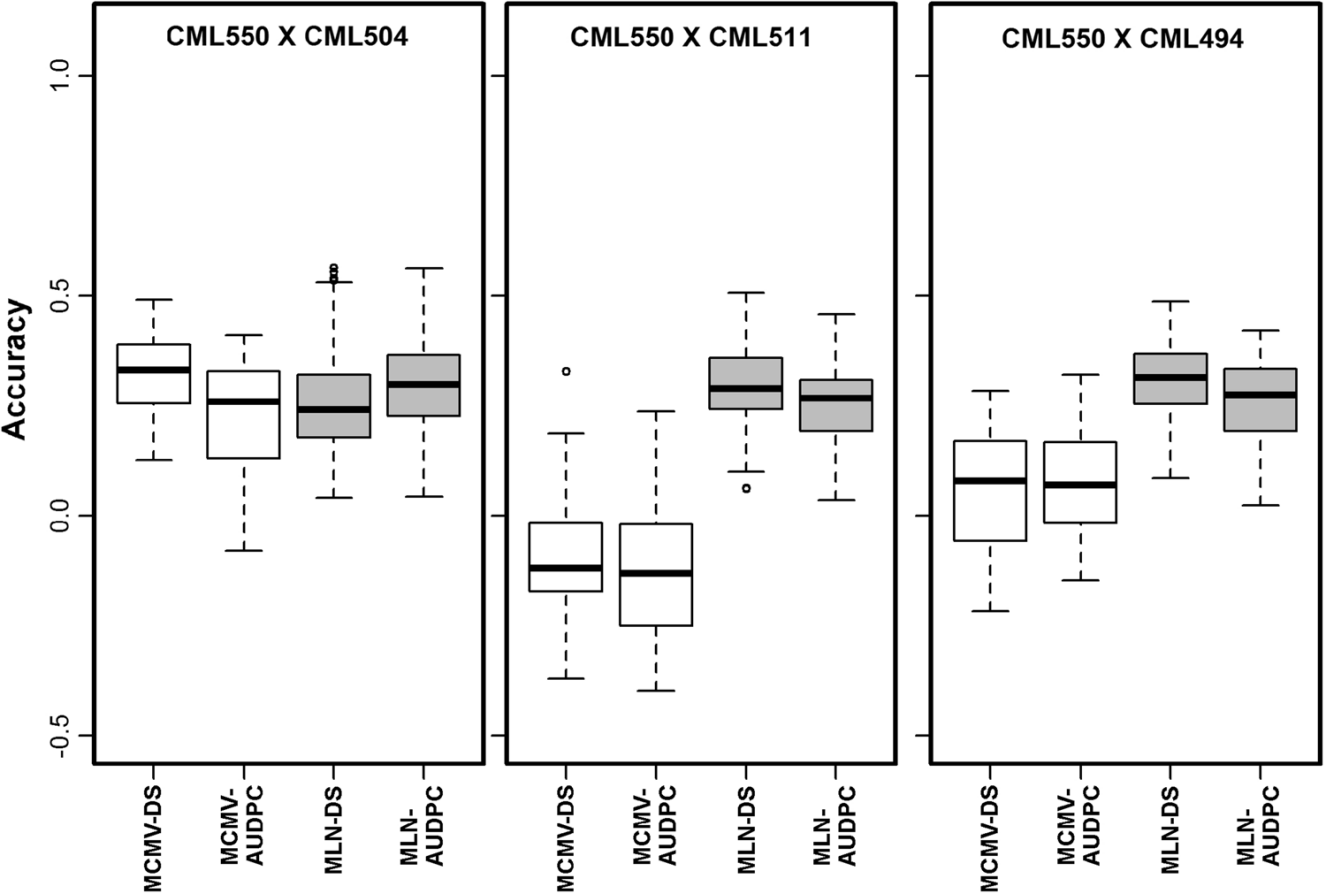
Accuracy of genomic predictions for effect estimation in the IMAS association panel and prediction in individual DH populations. Results are shown for the MCMV-DS, MCMV-AUDPC, MLN-DS and MLN-AUDPC scores

We used fivefold cross-validation to assess the accuracy of GP for MCMV and MLN. For within-population predictions, the average accuracies for the IMAS panel, DHpop1, DHpop2 and DHpop3 were 0.32, 0.78, 0.47 and 0.21 for MCMV-DS, and 0.31, 0.95, 0.44 and 0.29 for MCMV-AUDPC, respectively (Fig. 4). For MLN-DS, the respective mean accuracies were 0.52, 0.86, 0.46 and 0.62, and for MLN-AUDPC they were 0.58, 0.87, 0.46 and 0.66, (Fig. 4). Predictions generated by combining DH populations, and DH populations with the IMAS panel revealed significant improvement in the accuracy. Overall, the combined DH populations alone yielded higher accuracy than the DH populations combined with the IMAS panel (Fig. 4). In summary, the accuracies were consistently higher for MLN than for MCMV for both DS and AUDPC. For across-population predictions, the accuracy varied depending on the testing population and was even negative for DH pop2 (Fig. 5). Overall, the accuracies for across populations were substantially lower compared to within-population and combined-population-based predictions. Populations with high heritability and large population size showed high prediction accuracy compared to others.

## Discussion

Accurate phenotyping is fundamental for studying genetic architecture of traits, including genetic resistance to plant diseases. MLN, caused by co-infection of MCMV and SCMV, is a complex disease, seriously threatening food security in eastern Africa. Most of the elite inbred lines and commercial hybrids are susceptible to MCMV and MLN (De Groote et al. 2016). Breeding for MLN resistance is complicated, as one needs to phenotype accurately under artificial inoculation in isolated or controlled conditions; this process is cumbersome and labor intensive. The genetics of SCMV resistance has been studied more extensively (Tao et al. 2013; Gustafson et al. 2018) than the genetics of MCMV resistance, so identifying, validating and deploying molecular markers associated with MCMV and/or MLN resistance could increase the efficacy of breeding programs engaged in developing MLN-resistant tropical and subtropical maize germplasm. Indeed, introgression of MCMV and MLN resistance-associated markers into the breeding pipeline is the next priority of the maize breeding programs of CIMMYT as well as the National Agricultural Research System (NARS) partners in eastern Africa.

The distribution of lines in each of the mapping populations, as well as in combined populations, for the DS and AUDPC of MCMV and MLN (Fig. 1) suggests a polygenic nature for both MCMV and MLN resistance. Earlier studies on the inheritance of resistance to MCMV (Jones et al. 2018) and MLN (Gowda et al. 2015, 2018; Beyene et al. 2017) also confirmed polygenic control. In the current work, we observed significant genetic variances and moderate-to-high heritability for DS and AUDPC of both MCMV and MLN, indicating good prospects for breeding for resistance against MCMV and MLN in tropical maize germplasm. This closely parallels earlier studies of biparental populations of SCMV (Xia et al. 1999), MCMV (Jones et al. 2018) and MLN (Gowda et al. 2018), and association panels of SCMV (Leng et al. 2015; Gustafson et al. 2018) and MLN (Gowda et al. 2015). MLN is due to the individual effects of SCMV and MCMV, as well as their interaction effects. Selection for MCMV resistance can improve resistance against MLN, which is also well supported by the significant positive correlations we observed in this study (correlation range *r* = 0.33 to 0.60) between MCMV and MLN for both DS and AUDPC in all populations except DH pop3 (data not shown). However, undertaking screening and breeding for resistance to individual viruses could be more laborious than breeding for MLN resistance as a single trait.

QTL analyses in the three DH populations revealed a genomic region in chromosome 3, between 85 and 109 mega base pairs (Mbp), as being important for both MCMV and MLN resistance. One major QTL, *qMCMV3*-*108*, identified in this region is very consistent with the DS scores and AUDPC values in both DH pop1 and DH pop2 for both MCMV and MLN (Table 2). Further, this QTL also explained the highest proportion of variance of up to 58% for MCMV-AUDPC. An earlier GWAS on MLN (Gowda et al. 2015) revealed the three SNPs *S3_90976749, S3_90976758,* and *S3_114355785,* which fell within the same confidence interval as *qMCMV3*-*108* QTL. This QTL is also consistent with the previously reported MLN QTL, *qMLN_03*-*129*, identified in multiple biparental populations (Gowda et al. 2018). The physical position of the major SCMV QTL, *SCMV2*, is ~ 133 Mbp on chromosome 3 (Gustafson et al. 2018). The previous multiple population study (Gowda et al. 2018) and the results of the present study suggest that the genomic region between 100 and 119 Mbp in chromosome 3 is important for MCMV and MLN resistance. This genomic region seems to be different from the *Scmv2* QTL. *qMCMV4*-*235* is another consistent QTL detected for both DS and AUDPC of MCMV as well as for MLN (Table 2). This QTL is also consistent with a previously reported QTL for MLN in an F_3_ population (Gowda et al. 2018). This implies that the detected major QTL is associated specifically with MCMV resistance and useful for improving MCMV resistance and ultimately MLN resistance. In contrast, three quantitative trait nucleotides (QTNs) identified for SCMV resistance in a diversity panel (Gustafson et al. 2018) fell within the confidence interval of the three MCMV resistance QTLs, *qMCMV1*-*290, qMCMV2*-*192* and *qMCMV4*-*235*, which supports the clustering nature of viral disease resistance genes in maize (Zambrano et al. 2014). Furthermore, there were 11 new QTLs that were identified specifically for MCMV across the three DH populations in the current study; this clearly points out the complex nature of MCMV resistance.

Among the 11 QTLs detected for MLN in three DH populations, eight of QTLs were found only with MLN and they were not detected for MCMV resistance (Table 2). Nevertheless, the QTL detected on chromosome 6, *qMLN6*-*158*, overlaps with the QTL reported for MCMV resistance in F_2_ populations (Jones et al. 2018). Surprisingly, in the same genomic region, a QTL for SCMV resistance was also reported from a diversity panel (Gustafson et al. 2018). Another QTL, *qMLN6*-*89*, overlaps with MLN resistance QTL reported in F_3_ populations (Gowda et al. 2018). Major-effect QTLs for SCMV are known to be present on chromosome 3 (133 Mbp) and chromosome 6 (13–16 Mbp). Interestingly, these eight MLN-specific QTL also did not fall into major SCMV QTL regions, which warrants further research/validation to understand whether these QTLs respond to SCMV or MCMV separately or respond to their interactions. This could help breeders design effective strategies for introgressing such QTLs into breeding materials through marker-assisted breeding.

In DH pop1, the major QTL, *qMCMV*-*108/qMLN3*-*108*, identified on chromosome 3 with a LOD score of 53.9 and explaining up to 58% of the phenotypic variation for MCMV and 23% for MLN indicated that CML 550 is a source of favorable alleles (Fig. 2). The segregation alleles from two tightly linked markers for this major QTL reveal that DH lines and inbred lines from the IMAS panel with low DS scores were strongly associated with alleles from CML550, the MCMV- and MLN-tolerant parent (Fig. 2). Thus, CML550 can be used as a potential trait donor to introgress the major QTL identified on chromosome 3. This finding also agrees with Beyene et al. (2017) who reported CML550 as one of the best lines with high general combining ability (GCA) for MLN tolerance. Nevertheless, as part of validation, the efficiency of these flanking markers should be assessed further through KASP (Kompetitive allele specific PCR) assays, where we can check their ability to identify MLN- and MCMV-resistant and susceptible genotypes.

JLAM was implemented with the aim of taking advantage of both its high QTL detection power and improved resolution to robustly identify MCMV and MLN resistance QTLs. In line with this expectation, the consistent and major-effect QTL, *qMCMV3*-*108/qMLN3*-*108*, identified with confidence intervals of 67Mbp (51–119 Mbp) in DH pop2 and 24 Mbp (86–109 Mbp) in DH pop1 was reduced and five significant markers identified within this region. Two markers, *S3_108706910 (qMCMV3*-*108)* and *S3_116124132 (qMCMV3*-*116)*, were identified with MCMV resistance and three markers, *S3_86873766 (qMLN3*-*86), S3_87781149 (qMLN3*-*87)* and *S3_119614021(qMLN3*-*119)*, were significantly associated with MLN resistance (Tables 2, 3). This suggests the possibility of more than one QTL in this region. *S3_119614021(qMLN3*-*119)* was also reported as an important genomic region for MLN resistance (Gowda et al. 2018) and appears to be different from the major-effect QTL *SCMV2* (133 Mbp), which was also detected in the same study.

JLAM exploits variations from across and within populations and is able to detect new QTLs that might be missed in linkage mapping. In accordance with this observation, we found some new QTLs that were not detected by individual population-based QTL mapping, most notably *S6_17165743* (*qMCMV6*-*17/qMLN6*-*17*). The *S6_17165743* marker was identified close to the *Scmv1* region, a key gene for SCMV resistance; interestingly in addition to MLN resistance, the same *S6_17165743* QTL (*qMCMV6*-*17/qMLN6*-*17*) was also significantly associated with MCMV resistance and explained 27.2% of phenotypic variance (Table 3).

SCMV is an important component of MLN in eastern Africa. *Scmv1* is a major gene for SCMV resistance. However, in this study we did not find the same QTL associated with MLN resistance in the DH populations. This is probably due to complete absence of markers in this region; markers were removed due to a high missing rate in many lines, especially in the region between 12 and 16 Mbp on chromosome 6. A large presence–absence variation has been reported in this region on the short arm of chromosome 6 where the *Scmv1* is known to be present. This is similar to the observations of Tao et al. (2013) and Gustafson et al. (2018) on their association panel where they were unable to amplify any markers in this region in many lines. Nevertheless, *S6_17165743* is an important QTL for MLN resistance and possibly contributes toward both MCMV and SCMV resistance. Overall, JLAM efficiency was improved significantly due to increased population size, allele diversity and balanced allele frequencies.

As maize has high genetic diversity and rapid LD decay, GWAS has been commonly used to analyze the genetic architecture of many complex diseases. In the present study, we found 13 SNPs associated with MCMV resistance and 39 SNPs with MLN resistance (Table 4 and Supplementary Table S3). All the identified SNPs seemed to have minor effects, as revealed by the PVE by each SNP for both MCMV and MLN. Even though MLN is due to a combination of MCMV and SCMV, in this study we were not able to find common SNPs across MCMV and MLN. Some of the identified SNPs with MCMV and MLN resistance showed strong functional association with disease resistance genes, particularly related to the WRKY DNA-binding protein (Yu et al. 2001) and serine/threonine protein kinase (Lin et al. 2015). Nevertheless, GWAS results must be taken cautiously as we observed smaller deviation of SNPs toward expected *P* values in QQ plots which indicates the possibility of selection of false positives (Fig. 3). Therefore, the main-effect QTL detected not only through association panel but also detected in different populations should be considered for breeding applications.

Validation of QTLs detected through linkage mapping and JLAM through GWAS revealed some consistently overlapping genomic regions (Tables 2, 3, 4 and S3). This is very much clear in the integrated physical map where all the QTLs detected for MCMV and MLN in each DH population and JLAM, QTNs for GWAS were mapped (Supplementary Fig. S4). Specifically, SNP *S1_79444916* detected for MCMV resistance was within the confidence interval (CI) of the QTL *qMCMV1*-*10* and located very close (< 2 Mbp) to the other two QTLs, *qMCMV1*-*76* and *qMCMV1*-*80*, detected in linkage mapping. QTL *qMCMV1*-*71* found through JLAM co-occurred with SNP *S1_79444916* and *S8_149982735* is another marker that overlaps with QTL *qMCMV8*-*169*, which has a CI of 149–169 Mbp. *S7_130133358* is another SNP located within the CI of the QTL *qMCMV7*-*132*, whereas SNP *S2_197143379* is located close to the QTL *qMCMV2*-*192*. For MLN, six SNPs are located within the CI of QTL *qMCMV1*-*10* and five significant SNPs were identified within the CI of *qMLN3*-*167*. SNP *S6_158471262* on chromosome 6 was identified within the CI of *qMLN6*-*158* and there were four SNPs that were also identified in this region within < 5 Mb using JLAM, which suggests the importance of this region for MLN resistance. Jones et al. (2018) observed major recessive QTLs in this region in F_2_ populations, whereas Gustafson et al. (2018) also found QTLs for SCMV resistance in their association panel in the same region. In chromosome 7, we found two SNPs that fell within the CI of the QTLs *qMLN7*-*130* and *qMLN7*-*152* (Tables 3 and S2). Validation results suggest genomic regions identified in chromosomes 1, 3, 6 and 7 were consistent across DH populations and the GWAS panel, and they might have potential for marker-assisted breeding for MLN resistance.

Understanding the functional mechanism of genes involved in the stable QTL regions can establish a strong association between resistance gene candidates and both qualitative and quantitative resistances. In line with this expectation, in the *qMLN3*-*108* QTL region we found SNPs like *S3_51499448* associated with hydrogen peroxide detoxification, *S3_85659716* associated with leucine-rich repeat protein, *S3_109388419* associated with zinc ion binding function, and *S3_116124132* associated with WRKY DNA-binding protein, and in *qMLN3*-*17* region *S6_17165743* associated with D-amino acid aminotransferase which involved in a process where RNA molecules inactivate expression of target genes (https://phytozome.jgi.doe.gov/phytomine/results). An unusually high frequency of genes conferring recessive resistance is observed in interactions with potyviruses (Jones et al. 2018; Shi et al. 2005). Therefore, it warrants further research through candidate-gene approach on the stable QTL which can able to pinpoint the resistance QTLs as well as help to understand the molecular mechanisms underlying the development and progression of SCMV and MCMV infection and development of plant resistance in maize.

GP within populations showed high accuracy in DH pop1 and the combined DH populations for both MCMV and MLN, and this is encouraging for the use of GP in MCMV and MLN resistance breeding. The high accuracy in the DH populations is also due to their expected high LD blocks relative to the IMAS panel. In contrast, we found low accuracy in DH pop3 for MCMV, which could be due to its small range of variability within population as well as low heritability (Table 1, Fig. 4). The accuracy was moderate for both MCMV and MLN in the IMAS panel, which is explained by the broad genetic base of the panel (Gowda et al. 2015; Zhang et al. 2017). The observed differences in the accuracies in different populations studied here could be due to their differences in sample size, genetic variance, trait heritability, changes in population structure and LD estimates. Trait-wise comparison of accuracy reveals better predictions for MLN over MCMV (Fig. 4). Although MLN resistance is more complex than MCMV resistance, the observed difference in accuracy can be attributed to high genetic variation, heritability (Table 1) observed for MLN over MCMV and possibly the contribution of all the segregating major-effect QTLs in all populations for both MCMV and SCMV.

In breeding for resistance to MLN and MCMV, it is useful to have a common training population to reduce labor-intensive phenotyping. In this study, we have three DH populations whose parents are part of the association mapping panel and are highly related to several lines derived from subtropical breeding program. High relatedness among DH populations and IMAS association mapping panel is also evident with PCA (Supplementary Fig S3). For GP, we used the IMAS panel as a training population and the DH populations as testing populations (Fig. 5). This scenario was applied by considering relatively simple genetic architecture and high heritability for MLN and MCMV compared to complex traits like grain yield and practical scenarios of breeding. We observed reasonable accuracies, but these were lower than the prediction accuracies observed within populations and in the combined populations (Figs. 4, 5). Prediction accuracies also varied with testing populations. The negative accuracies observed for DH pop2 for MCMV are intriguing; similar results were also reported for prediction among less related biparental populations in maize (Riedelsheimer et al. 2013) and sugar beet (Würschum et al. 2013). Opposite linkage phases between markers and major-effect QTLs in the IMAS panel and DH pop 2 might be another possible explanation for negative accuracy. In addition, a lower magnitude of observed genotypic variation and low heritability for MCMV might also have contributed to lower prediction accuracy. In contrast, the prediction accuracies for MLN were similar for all three DH populations; this may be due to the major QTL being in the same linkage phase and segregating in both the IMAS panel and the DH populations as well. Overall, the obtained prediction accuracies, particularly for MLN resistance, are promising and showed that this approach does hold potential for application in breeding for MLN resistance.

In conclusion, we used three DH populations and one IMAS association mapping panel, together comprising 965 lines, to unravel the genetic architecture of MCMV resistance, and this approach identified new QTLs. In addition, we validated the reported QTLs for MLN resistance in tropical and subtropical maize germplasm. Linkage mapping identified two new major-effect QTLs that were consistent for both MCMV and MLN resistance. The detected QTLs were validated with GWAS, and several SNPs were found overlapping with the identified QTLs through either linkage mapping or JLAM. These genomic regions can serve as potential sources to improve resistance to MCMV and MLN. GP can be used within populations to predict the response of the germplasm to MCMV and MLN resistance. Having a common training population derived from intensively phenotyped and genotyped lines with diverse representation from a breeding program holds promise in breeding for MLN resistance.

### Author contribution statement

CS, SLM, YB, DM, KO, MSO, BMP, BD, JMB, SM, AT, JC, MG. Chelang’at Sitonik^1,3^, Suresh LM^1^, Yoseph Beyene^1^, Dan Makumbi^1^, Kiplagat Oliver^3^, Michael S Olsen^1^, Boddupalli M Prasanna^1^, Biswanath Das^1^, Jumbo M Bright^1^, Stephen Mugo^1^, Amsal Tarekegne^4^, Jose Crossa^2^, Manje Gowda^1*.^ BMP, MG and SLM conceived the project; CS, MG, SLM and YB carried out the experiments; BD, JMB and AT participated in field trials; MG, MSO and JMB performed genotyping; BD, DM and YB developed the DH populations; SC and MG performed phenotyping of the all populations; MG and JC carried out data analyses; MG, YB, DM, BD, MSO, JC, BMP, JMB, KO, SM and SLM interpreted the results and drafted the manuscript.

## Electronic supplementary material

Below is the link to the electronic supplementary material.

Supplementary material 1 (DOCX 656 kb)

## Abbreviations

GBS: Genotyping-by-sequencing
GP: Genomic prediction
JLAM: Joint linkage association mapping
LD: Linkage disequilibrium
MAF: Minor allele frequency
MCMV: *Maize chlorotic mottle virus*
MLN: Maize lethal necrosis
MLM: Mixed linear model
QTL: Quantitative trait locus
