# Supplementary material for: Genetic architecture of maize chlorotic mottle virus and maize lethal necrosis through GWAS, linkage analysis and genomic prediction in tropical maize germplasm
Source: Theor Appl Genet. 2019 May 16;132(8):2381–99. doi: 10.1007/s00122-019-03360-x (PMC6647133; doi:10.1007/s00122-019-03360-x)
Supplement: Supplementary file 1 — Supplementary material 1 (DOCX 656 kb) [file 122_2019_3360_MOESM1_ESM.docx]

**Supplementary Table S1** List of 12 best performing maize lines for MCMV resistance, and their disease severity scores for MLN**.**

| Inbreds | MCMV | | MLN | | Heterotic group |
| --- | --- | --- | --- | --- | --- |
|  | DS | AUDPC | DS | AUDPC |  |
| CML550 | 3.36 | 147.06 | 4.58 | 137.44 | B |
| LaPostaSeqC7-F71-1-2-1-1-B | 3.37 | 139.49 | 4.13 | 124.34 | A |
| (CML550/CML504)DH73 | 3.47 | 168.41 | 4.94 | 140.41 | B |
| (CML550/CML504)DH128 | 3.50 | 173.91 | 4.71 | 120.95 | B |
| DTPWC9-F31-1-3-1-1-B | 3.53 | 146.18 | 5.08 | 161.32 | A |
| (CML550/CML504)DH30 | 3.55 | 175.40 | 5.14 | 137.76 | B |
| CLWN276 | 3.55 | 140.71 | 4.99 | 163.84 | B |
| CML342 | 3.55 | 152.52 | 4.11 | 128.25 | AB |
| CML373 | 3.56 | 139.82 | 4.65 | 149.72 | A |
| (CML550/CML504)DH91 | 3.56 | 179.92 | 4.88 | 136.24 | B |
| (CML550/CML504)DH35 | 3.56 | 178.60 | 4.97 | 143.75 | B |
| (CML550/CML504)DH161 | 3.58 | 179.70 | 4.96 | 136.71 | B |

**Supplementary Table S2**. Summary of the linkage groups constructed for three DH populations.

| **Population** | **No of progenies** | **No of SNPs** | **Map length** | **Avg distance (cM)** |
| --- | --- | --- | --- | --- |
| CML550 X CML504 - DH pop1 | 219 | 931 | 2666.08 | 2.86 |
| CML550 X CML511 - DH pop2 | 111 | 929 | 2922.24 | 3.14 |
| CML550 X CML494 - DH pop3 | 229 | 940 | 2706.95 | 2.88 |

**Supplementary Table S3** Chromosomal position and SNPs significantly associated with MLN disease severity (DS) and Area under Disease Progress Curve (AUDPC) detected by SNP-based GWAS in the IMAS association mapping panel.

| **Trait** | **SNP-name** | **Chr** | **Position (bp)** | **MLM** | | **MAF** | **Allele^a^** | **Putative candidate gene** | **Predicted function of candidate gene** |
| --- | --- | --- | --- | --- | --- | --- | --- | --- | --- |
|  |  |  |  | **P values** | **R^2^** |  |  |  |  |
| **MLN disease severity** | S1_24941000 | 1 | 24941000 | 7.79E-06 | 0.066 | 0.28 | T/C | GRMZM2G032423 | Unknown |
|  | S1_148456035 | 1 | 148456035 | 5.58E-06 | 0.069 | 0.04 | T/A | GRMZM2G135045 | Manganese ion binding; proteolysis; aminopeptidase activity |
|  | S1_182356356 | 1 | 182356356 | 4.50E-05 | 0.063 | 0.43 | T/C | GRMZM2G379179 | Uncharacterized protein |
|  | S2_105760109 | 2 | 105760109 | 5.02E-05 | 0.057 | 0.12 | G/A | GRMZM2G137984 | Protein binding; retrograde transport endosome to Golgi |
|  | S2_184968171 | 2 | 184968171 | 1.40E-05 | 0.06 | 0.33 | T/A | GRMZM2G414252 | Uncharacterized protein; Transcription regulator activity |
|  | S2_193503877 | 2 | 193503877 | 3.83E-05 | 0.06 | 0.33 | G/C | GRMZM2G150503 | Unknown |
|  | S2_211771737 | 2 | 211771737 | 1.12E-05 | 0.063 | 0.02 | G/T | GRMZM2G169087 | Carbon-nitrogen ligase activity with glutamine as amido-N-donor |
|  | S3_34036135 | 3 | 34036135 | 1.37E-05 | 0.065 | 0.14 | G/T | GRMZM2G094523 | Uncharacterized protein; plant-type cell wall organization; sugar binding |
|  | S3_44062810 | 3 | 44062810 | 1.60E-05 | 0.062 | 0.33 | T/C | GRMZM2G404316 | Ice binding; response to freezing |
|  | S3_114355785 | 3 | 114355785 | 2.65E-05 | 0.057 | 0.2 | T/C | GRMZM2G405385 | response to freezing; ice binding |
|  | S3_165904362 | 3 | 165904362 | 2.84E-05 | 0.064 | 0.07 | A/G | GRMZM2G177198 | Hypothetical protein |
|  | S3_189356738 | 3 | 189356738 | 5.14E-05 | 0.055 | 0.27 | A/C | GRMZM2G396029 | Unknown |
|  | S5_5205032 | 5 | 5205032 | 3.75E-06 | 0.07 | 0.39 | T/A | GRMZM5G892151 | Unknown |
|  | S5_182137972 | 5 | 182137972 | 4.62E-05 | 0.056 | 0.1 | A/G | GRMZM2G137375 | Uncharacterized protein |
|  | S5_198047758 | 5 | 198047758 | 2.32E-05 | 0.059 | 0.15 | C/T | GRMZM2G428242 | Pathogenesis; ATP binding; Helicase activity; DNA binding |
|  | S5_198047802 | 5 | 198047802 | 3.26E-05 | 0.06 | 0.14 | T/A | GRMZM2G428242 | pathogenesis; ATP binding; Helicase activity; |
|  | S7_2874978 | 7 | 2874978 | 5.06E-05 | 0.06 | 0.42 | C/T | GRMZM2G067946 | freezing; G-protein coupled receptor protein signaling pathway; vasopressin receptor activity |
|  | S7_115310293 | 7 | 115310293 | 1.14E-05 | 0.063 | 0.17 | T/C | GRMZM2G125653 | WRKY76-superfamily of TFs having WRKY and zinc finger domains; regulation of transcription; |
|  | S7_159410999 | 7 | 159410999 | 4.51E-05 | 0.06 | 0.14 | C/G | GRMZM5G816386 | Unknown |
|  | S8_82856421 | 8 | 82856421 | 2.57E-05 | 0.063 | 0.02 | A/C | GRMZM2G055116 | Uncharacterized protein |
|  |  |  |  |  |  |  |  |  |  |
| **Trait** | **SNP-name** | **Chr** | **Position (bp)** | **MLM** | | **MAF** | **Minor Allele** | **Putative candidate gene** | **Predicted function of candidate gene** |
|  |  |  |  | **P values** | **R^2^** |  |  |  |  |
| **MLN AUDPC value** | S1_18630252 | 1 | 18630252 | 1.62E-05 | 0.06 | 0.12 | C/T | GRMZM2G398848 | Putative uncharacterized protein |
|  | S1_24941000 | 1 | 24941000 | 7.79E-06 | 0.07 | 0.28 | T/C | GRMZM2G032423 | Unknown |
|  | S1_52083545 | 1 | 52083545 | 3.16E-05 | 0.06 | 0.20 | A/G | GRMZM2G138814 | SNF1-related protein kinase regulatory subunit beta-1 |
|  | S1_199485008 | 1 | 199485008 | 1.20E-05 | 0.07 | 0.39 | G/A | GRMZM2G478417 | BZIP transcription factor ABI5 ; transcription factor activity |
|  | S1_230797791 | 1 | 230797791 | 4.12E-05 | 0.06 | 0.10 | C/T | GRMZM2G069976 | Putative uncharacterized protein; Hypothetical protein |
|  | S2_8916323 | 2 | 8916323 | 5.27E-05 | 0.06 | 0.02 | A/T | GRMZM2G076239 | Hydroxyacid oxidase 1;oxidoreductase activity |
|  | S2_51229897 | 2 | 51229897 | 2.86E-05 | 0.07 | 0.37 | T/C | GRMZM2G131177 | Serine-type endopeptidase activity |
|  | S2_104511943 | 2 | 104511943 | 1.97E-05 | 0.06 | 0.05 | G/C | GRMZM2G079616 | Transferase activity transferring acyl groups other than amino-acyl groups |
|  | S2_155536752 | 2 | 155536752 | 2.74E-05 | 0.06 | 0.10 | T/C | GRMZM2G703211 | Gamma-glutamyltransferase activity |
|  | S2_193503877 | 2 | 193503877 | 2.77E-05 | 0.06 | 0.33 | G/C | GRMZM2G150503 | Unknown |
|  | S3_22944526 | 3 | 22944526 | 3.33E-05 | 0.06 | 0.39 | C/T | GRMZM2G471517 | Response to freezing; ice binding; homoiothermy |
|  | S3_44062810 | 3 | 44062810 | 1.60E-05 | 0.06 | 0.34 | T/C | GRMZM2G404316 | Response to freezing; ice binding; homoiothermy |
|  | S3_44224185 | 3 | 44224185 | 2.56E-05 | 0.06 | 0.45 | G/A | GRMZM2G003883 | Pyruvate kinase; potassium ion binding; glycolysis |
|  | S3_45037427 | 3 | 45037427 | 2.84E-05 | 0.06 | 0.36 | G/A | GRMZM2G078895 | Putative uncharacterized protein |
|  | S3_114355785 | 3 | 114355785 | 2.87E-05 | 0.06 | 0.21 | T/C | GRMZM2G405385 | Response to freezing; ice binding; homoiothermy |
|  | S5_5205032 | 5 | 5205032 | 3.75E-06 | 0.07 | 0.39 | T/A | GRMZM5G892151 | Unknown |
|  | S5_198047758 | 5 | 198047758 | 2.32E-05 | 0.06 | 0.16 | C/T | GRMZM2G428242 | Pathogenesis; ATP binding; helicase activity |
|  | S6_158471262 | 6 | 158471262 | 5.46E-05 | 0.05 | 0.1 | A/C | GRMZM2G435445 | Putative uncharacterized protein |
|  | S7_2874978 | 7 | 2874978 | 1.84E-05 | 0.07 | 0.43 | C/T | GRMZM2G067946 | Response to freezing; G-protein coupled receptor protein signaling pathway; vasopressin receptor activity |
|  | S7_135837858 | 7 | 135837858 | 4.88E-05 | 0.06 | 0.79 | C/G | GRMZM2G128121 | catalytic activity |
|  | S7_140411743 | 7 | 140411743 | 3.77E-05 | 0.06 | 0.10 | C/T | GRMZM2G071015 | Unknown |
|  | S7_158464599 | 7 | 158464599 | 2.68E-05 | 0.06 | 0.23 | A/C | GRMZM2G006942 | Putative uncharacterized protein |
|  | S7_170395340 | 7 | 170395340 | 3.88E-05 | 0.06 | 0.28 | G/T | GRMZM2G019666 | oxidation reduction; heme binding; iron ion binding |
|  | S8_82856421 | 8 | 82856421 | 1.40E-05 | 0.07 | 0.03 | A/C | GRMZM2G055116 | Putative uncharacterized protein |
|  | S9_11775297 | 9 | 11775297 | 1.89E-05 | 0.06 | 0.38 | C/A | GRMZM2G165390 | Anthocyanidin 3-O-glucosyltransferase |
|  | S10_23785810 | 10 | 23785810 | 2.13E-05 | 0.06 | 0.03 | G/A | GRMZM2G451231 | microtubule-based movement; ATP binding; microtubule motor activity |
|  |  |  |  |  |  |  |  |  |  |

MAF minor allele frequency, **^a^** Minor allele is underlined, R^2^ proportion of phenotypic variance explained, MLM Mixed linear model

^a^The exact physical position of the SNP can be inferred from marker’s name, for example, S1_24941000: chromosome 1; 24,941,000 bp


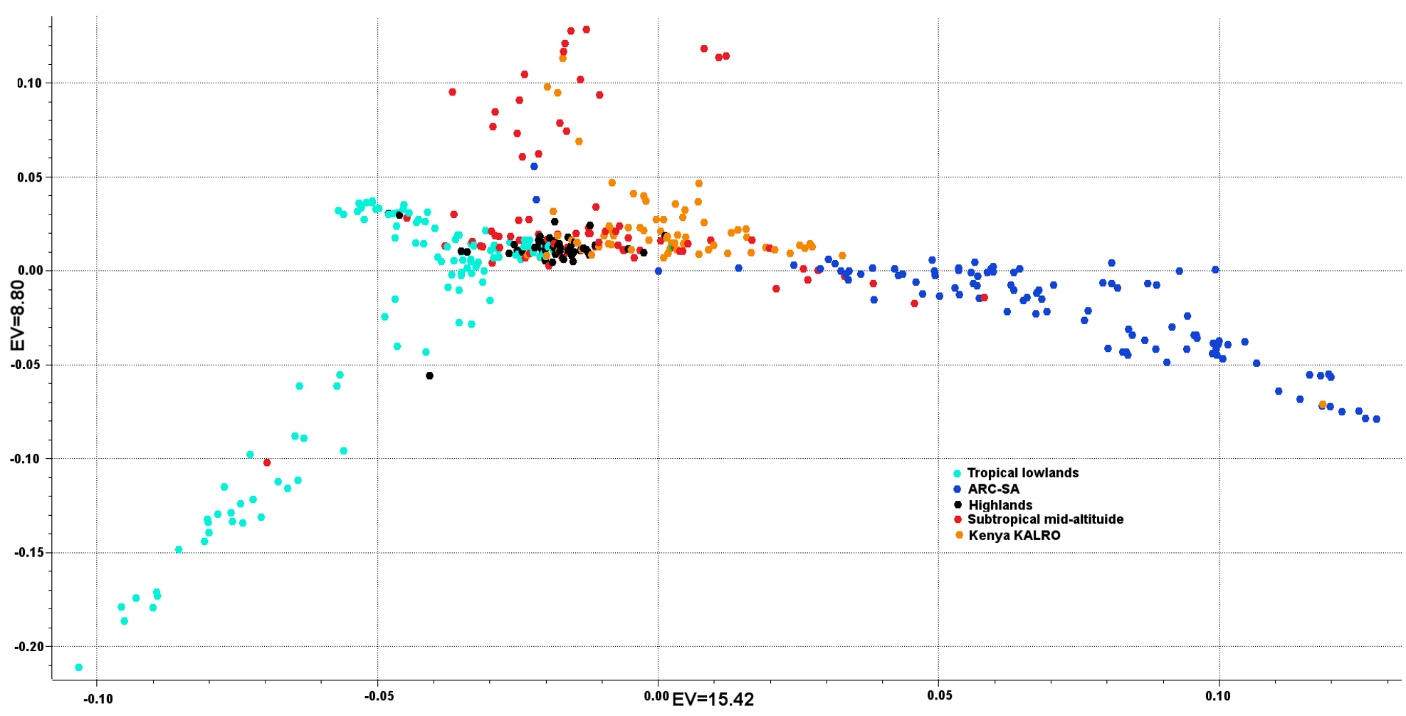


**Supplementary Figure S1**: Population structure based on principal component (PC) analysis of IMAS association mapping panel


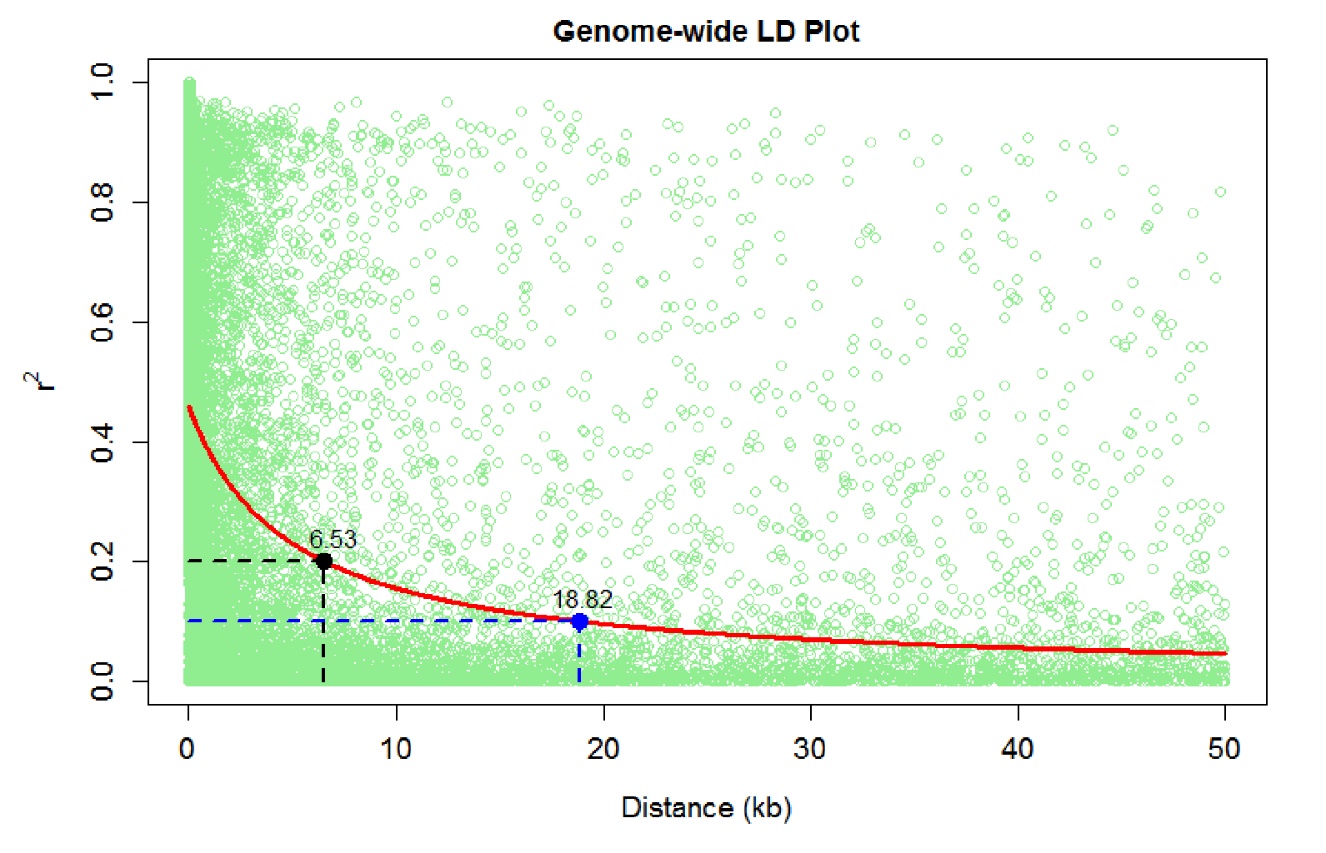


**Supplementary Figure S2.** Linkage disequilibrium (LD) plot representing the average genome-wide LD decay of the IMAS panel. The values on the Y-axis represent the squared correlation coefficient (r^2^) and the X-axis represents the genetic distance in kilobases (Kb). The blue and black dashed lines show the LD threshold for the association panel at r^2^=0.1 and 0.2, respectively.


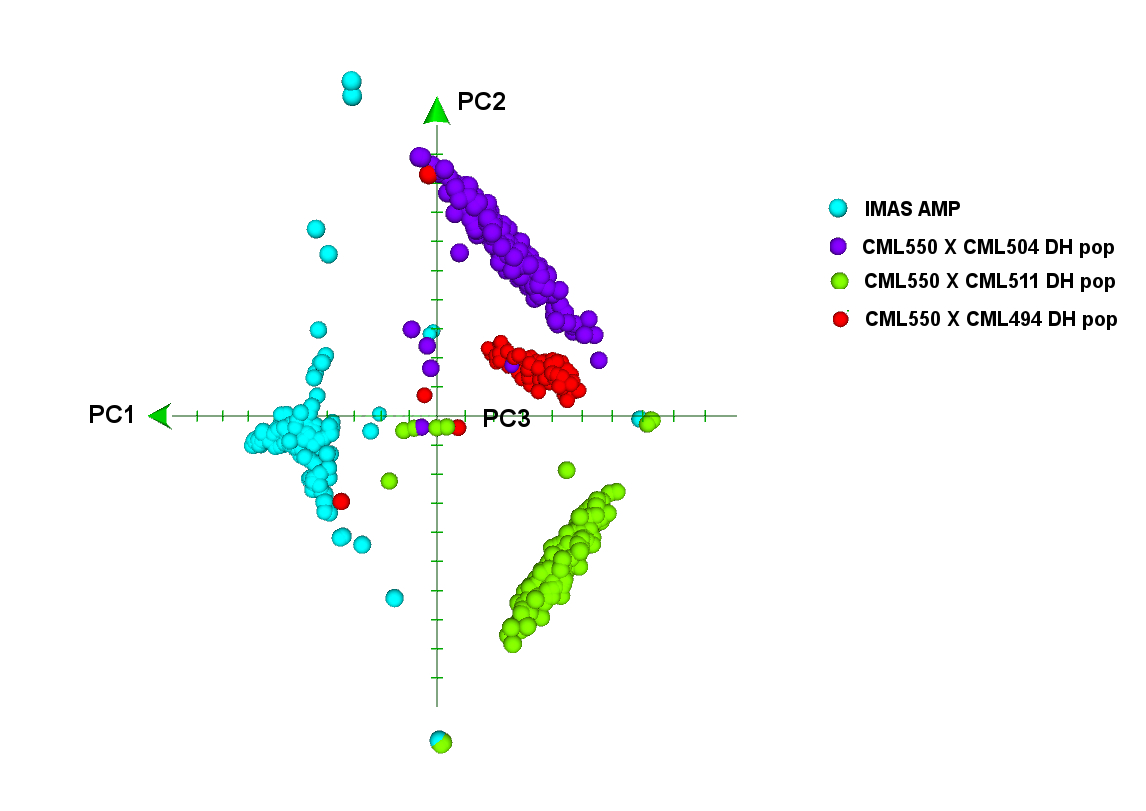


**Supplementary Figure S3.** Principal component (PC) analysis across IMAS association mapping panel and three DH populations.

**Supplementary Figure S4:** Integrated physical map and positioning of the QTL detected in three DH populations and joint linkage association mapping and IMAS association mapping for MCMV and MLN resistance. The number in the left side of the map are the distance in Mbp. QTL name composed by the trait code followed by the chromosome number in which the QTL was mapped and a physical position of the QTL. QTL detected for MCMV and MLN were marked with green and red color, respectively. Pop1 – CML550 x CML504; Pop2 – CML550 x CML511; Pop3 – CML550 x CML494; JLAM – Joint linkage association mapping; GWAS – Genome-wide association mapping

.
